# *NTR1* is involved in heat stress tolerance through mediating expression regulation and alternative splicing of heat stress genes in *Arabidopsis*

**DOI:** 10.3389/fpls.2022.1082511

**Published:** 2023-01-10

**Authors:** Lei He, Qi Wu, Ye Jin, Ye Fan, Huazhong Shi, Yizhong Wang, Wannian Yang

**Affiliations:** ^1^ School of Life Sciences, Central China Normal University, Wuhan, Hubei, China; ^2^ Department of Chemistry and Biochemistry, Texas Tech University, Lubbock, TX, United States

**Keywords:** heat stress (HS), heat stress tolerance, NTC-related protein 1 (NTR1), HS response (HSR) genes, alternative splicing (AS), intron-lariat spliceosome (ILS) complex

## Abstract

As a common adverse environmental factor, heat stress (HS) not only drastically changes the plant transcriptome at the transcription level but also increases alternative splicing (AS), especially intron retention (IR) events. However, the exact mechanisms are not yet well understood. Here, we reported that NTC-related protein 1 (NTR1), which acts as an accessory component for spliceosome disassembly, is necessary for this process. The mutants of *NTR1*, both the T-DNA insertion and the point mutation identified through ethyl methanesulfonate (EMS) mutagenesis screening, are vulnerable to HS, indicating that *NTR1* is essential for plant HS tolerance. At the molecular level, genes of response to heat and response to temperature stimulus are highly enriched among those of heat-induced but less-expressed *ntr1* mutants. Moreover, a large portion of HS response (HSR) genes such as heat shock transcription factors (HSFs) and heat shock proteins (HSPs) are less induced by heat treatment, and more AS events, especially IR events, were found in heat-treated *ntr1* mutants. Furthermore, HS suppressed the expression of *NTR1* and NTR1-associated complex components. Thus, it is very likely that upon HS, the plant reduces the expression of the NTR1*-*associated complex to fulfill the fast demands for transcription of HSR genes such as HSFs and HSPs, which in turn results in the accumulation of improperly spliced especially IR products and eventually causes harm to plants.

## Introduction

As global warming has accelerated in recent decades and extremely high temperature frequently happens, heat stress (HS) becomes a more and more adverse factor to affect plant growth and development, especially the yield of crops (Dusenge et al., 2019; Jagadish et al., 2021). HS influences the physiological and biochemical processes of plants such as impairing photosynthetic activity, respiration, and water metabolism (Zhao et al., 2020; Jagadish et al., 2021). Being sessile, plants evolved exquisite systems to counteract these unfavorable conditions and exhibit certain HS tolerance. Plants respond quickly to HS with new proteome or metabolites produced, such as molecular chaperones and antioxidant enzymes, achieving some extent of HS resistance (Jespersen, 2020).

At the molecular level, when encountering HS, plant cells reprogram the transcriptome mainly through the heat shock transcription factor (HSF)–heat shock protein (HSP) regulons. Plant HSFs are stimulated by HS and play a central role in HS response to regulate the expression of downstream HS response (HSR) genes such as HSPs. For example, HSFA1s act as master regulators in *Arabidopsis* since they activate the expression of other transcription factors (TFs) like *DEHYDRATION-RESPONSIVE ELEMENT BINDING PROTEIN 2A* (*DREB2A*), *HsfA2*, *HsfA7a*, *HsfBs*, and *MULTIPROTEIN BRIDGING FACTOR 1C* (*MBF1C*), and *hsfa1s* quadruple mutants (*hsfa1a*/*hsfa1b*/*hsfa1d*/*hsfa1e*) exhibit a severe decrease of thermotolerance and reduced induction of HSR genes (Yoshida et al., 2011; Ohama et al., 2017). *HsfA2* is steeply induced by HS and is needed for the extension of acquired thermotolerance (AT) (Charng et al., 2007; Lamke et al., 2016). In *Arabidopsis*, HsfB1 was reported as a repressor to repress expression of HSFA2, A7a, B1, and B2b (Ikeda et al., 2011). However, *hsfb1 hsfb2b* double mutant shows decreased AT than the wild type (Ikeda et al., 2011), indicating that HsfBs also promote HSR in *Arabidopsis*.

HSPs are chaperone proteins induced by HS as well as other stresses. They not only function for the renature of misfolded proteins caused by stress but also are involved in the post-translational regulation of HSFs. For instance, HSP70 and HSP90 could interact with HsfA1s through the temperature-dependent repression (TDR) domain of HsfA1s to repress the transactivation activity and nuclear localization of HsfA1 (Hahn et al., 2011). During HS, the increasing misfolded proteins cause dissociation of HSP70/HSP90 from HsfA1s, releasing HsfA1s to translocate to nuclear to activate downstream TFs (Ohama et al., 2016).

Non-coding RNAs are also engaged in HSR regulation. MiR398 is a direct target of *HsfA1* and is rapidly induced by HS. Targets of miR398 are reactive oxygen species (ROS)-scavenging genes. Induced miR398 results in ROS accumulation and then activation of *HsfA1*, which forms a positive feedback loop (Guan et al., 2013). MiR156 is also induced by HS and regulates HS memory through the miR156-SPL (SQUAMOSA-PROMOTER BINDING-LIKE) module (Stief et al., 2014; Zhao et al., 2016). Targets of ta-siRNAs (TRANS-ACTING SMALL INTERFERING RNAs) and HTT1/2 (HEAT-INDUCED TAS1 TARGET 1/2) are activated by HsfA1a and induced by HS. However, overexpression of TAS1a results in a decrease in thermotolerance as the expression of TAS1-siRNAs increases, resulting in reduced expression of HTT1/2 (Li et al., 2014).

Alternative splicing (AS), utilizing alternative splicing sites to form different mature mRNAs, is a key process for eukaryotes to enrich gene-encoded products and also an important way to regulate gene functions. Stress promotes AS events in plants, which result in the accumulation of dysfunctional products (Laloum et al., 2018). Moreover, AS may perform a vital role in the regulation of key players for plant stress response (Ling et al., 2021). For instance, severe HS causes HsfA2 to generate a splicing variant, which encodes a small truncated HsfA2 isoform (S-HsfA2). S-HsfA2 is only detected during HS or the recovery period after HS and functions as an HSF to activate HsfA2 expression (Liu et al., 2013). HS induces the production of a DREB2A variant transcript to form a truncated protein without the C-terminal domain required for DREB2A degradation (Vainonen et al., 2012). bZIP60, a basic leucine-zipper domain-containing transcription factor, is associated with unfolded protein response (UPR) in *Arabidopsis*. HS-induced UPR causes AS of bZIP60 to remove a 23-bp sequence, resulting in the translocation of bZIP60 from the endoplasmic reticulum (ER) into the nucleus (Deng et al., 2011; Ding et al., 2020). Furthermore, HS causes more AS events especially intron retention (IR) events globally compared to normal conditions in high plants, which may result from HS-induced obstacle of pre-mRNA splicing, or be an adaption to HS (Ling et al., 2018; Ling et al., 2021). The components of the splicing machinery itself are also regulated by AS during HS, which is mainly seen from reports of splicing factors of serine/arginine-rich (SR) proteins, that bind to RNA and facilitate pre-mRNA splicing (Ling et al., 2021). For instance, HS induces AS of SR30 to produce more mRNA variants that encode the full-length SR30 protein (Filichkin et al., 2010). HS not only promotes the transcription of SR45a but also increases exon skipping (ES) of the fifth exon of SR45a to form a full-length SR45a protein with an intact C-terminal that contains an arginine-serine repeat (RS) domain (Gulledge et al., 2012). However, how HS leads to the increase of AS especially IR events in high plants and whether other components or accessories of the spliceosome are involved in the regulation of HSR are not clear yet.

NTR1 (NTC-related protein 1), also named SPLICEOSOMAL TIMEKEEPER LOCUS1 (STIPL1), is an accessory component involved in spliceosome disassembly and evolutionally conserved between animals and plants (Dolata et al., 2015). In plants, NTR1 together with Increased Level of Polyploidy1-1D (ILP1), the DEAH-box-containing RNA helicase PRP43, PRE-MRNA PROCESSING 8 (PRP8), small nuclear RNAs (snRNAs), or other components forms the intron-lariat spliceosome (ILS) complex, which is dismantled in the last stage of the spliceosome cycle for recycling the splicing factors (Wang et al., 2019). In *Arabidopsis*, NTR1 is crucial for circadian rhythm regulation since mutation of *NTR1* causes AS splicing of circadian-associated genes (Jones et al., 2012). Additionally, NTR1 promotes microRNA (miRNA) biogenesis with ILP1 by assisting the transcription and splicing of MIRNA (MIR) genes in *Arabidopsis* (Wang et al., 2019). Here, we report that *NTR1* plays an essential role in promoting plant HS tolerance, as *NTR1* mutation results in mis-expression and increased false splicing of HSR genes, which contribute to the more vulnerable phenotype to HS for *ntr1* mutants, and HS suppresses the expression of NTR1-associated complex.

## Materials and methods

### Plant materials and growth conditions

T-DNA mutants of *NTR1* and their homolog gene *STPL2* are referred to as salk_073187c (*ntr1-1*) (Dolata et al., 2015) and cs315805 (*stpl2-T*) (Jones et al., 2012), and *ILP1* is referred to as salk_030650c (*ilp1-1*). The construction of an ethyl methanesulfonate (EMS) mutagenesis pool was described previously (Shen et al., 2016). Mutant *hl761* was obtained from screening the EMS mutagenesis pool of *pHSP18.2:LUC* in Col-0 with increased LUC activity after heat treatment. The background *pHSP18.2:LUC* in the Col-0 transgenic line referred to the control plants (CK). Plant seeds were sowed to half Murashige and Skoog (MS) solid medium to germinate and then transferred to soil for growth in the greenhouse under light:dark (LD) photoperiod with 16-h light and 8-h dark at 22°C to 25°C.

### Heat stress treatments

To score the phenotypes of HS tolerance, 7 days after germination (DAG) seedlings on half MS plates were treated with continuous heat exposure (38°C) for 16 h in the incubator (MIR-154, SANYO, Tokyo, Japan) and then moved to 22°C to recover for 5 days. Seedlings grown at 22°C for the same time duration were used for negative control.

### Luciferase imaging assays

The 7-DAG seedlings on half MS plates were treated for 1 h at 38°C first, and then the LUC activities for plants were imaged using a charge-coupled device (CCD) camera (DU934-BV, ANDOR Technology, South Windsor, CT, USA) after being sprayed with 1 mM of d-luciferin in 0.1% Triton X-100. The LUC signals were captured and processed using the Andor SOLIS software.

### RNA extraction and RT-qPCR

RNA was extracted from 10-DAG seedlings on half MS plates. For heat treatment, 10-DAG seedlings were treated at 38°C for the designed time period, collected, and then flash-frozen in liquid nitrogen for RNA extraction by using TRIzol reagent (Invitrogen, Carlsbad, CA, USA). For semi-quantitative PCR and RT-qPCR, cDNAs were synthesized using Prime Script™ RT reagent Kit with gDNA Eraser (Takara, Maebashi, Japan). RT-qPCR was performed on an ABI Prism 7900HT sequence detection system using AceQqPCR SYBR Green Master Mix (High ROX Premixed) Q141 (Nanjing, China), and *TUB2* (*AT5G62690*) was used as the internal control. The primers used are listed in **Table S2**.

### RNA-seq and analysis

For the RNA-seq experiment, the 10-DAG seedlings of *hl761* mutants and the control plants (CK) were used as materials. Seedlings were collected at three time points of heat treatment (38°C), which were before heat treatment and 15-min and 60-min heat treatment. Two biological replicates were used for analysis. Total RNA was extracted using the RNeasy Plus Mini Kit (Qiagen, Hilden, Germany) following the manufacturer’s instructions. The RNAs were sequenced by Illumina HiSeq 2500 platform at the company Annoroad (Beijing, China). RNA-seq reads were aligned to the *Arabidopsis* reference genome (TAIR10) using HISAT2 (Kim et al., 2019), and gene counts were determined using HTSeq (Anders et al., 2015). The differentially expressed genes (DEGs) were identified with Bioconductor package DESeq2 (Love et al., 2014) in R language with a fold change ≥1.5 or ≤2/3 with a false discovery rate (FDR) ≤0.05. Gene Ontology (GO) enrichment analysis was analyzed using agriGO (Tian et al., 2017). For differential AS events, analysis was performed with rMATS (with a threshold of 0.05 and false discovery rate ≤0.05) (Shen et al., 2014).

### Histological analysis of β-glucuronidase staining

About 10-day-old seedlings of the transgenic plants expressing *pNTR1:GUS* grew on half MS medium and were used as material. Before harvest, plants were heat treated at 38°C for 1 h or kept at 22°C as control. Then, seedlings were incubated in 90% acetone on ice for about 30 min and stained in β-glucuronidase (GUS) staining solution (100 mM of phosphate buffer, pH 7.0, 0.5 mM of potassium ferrocyanide, 0.5 mM of potassium ferricyanide, 0.1% Triton X-100, and 10 mM of EDTA, pH 8.0, 10% methanol, and 1 mM of X-gluc). The GUS staining levels were quantified with ImageJ.

## Results

### The identification of *NTR1* mutant *hl761*

**Figure 1:**
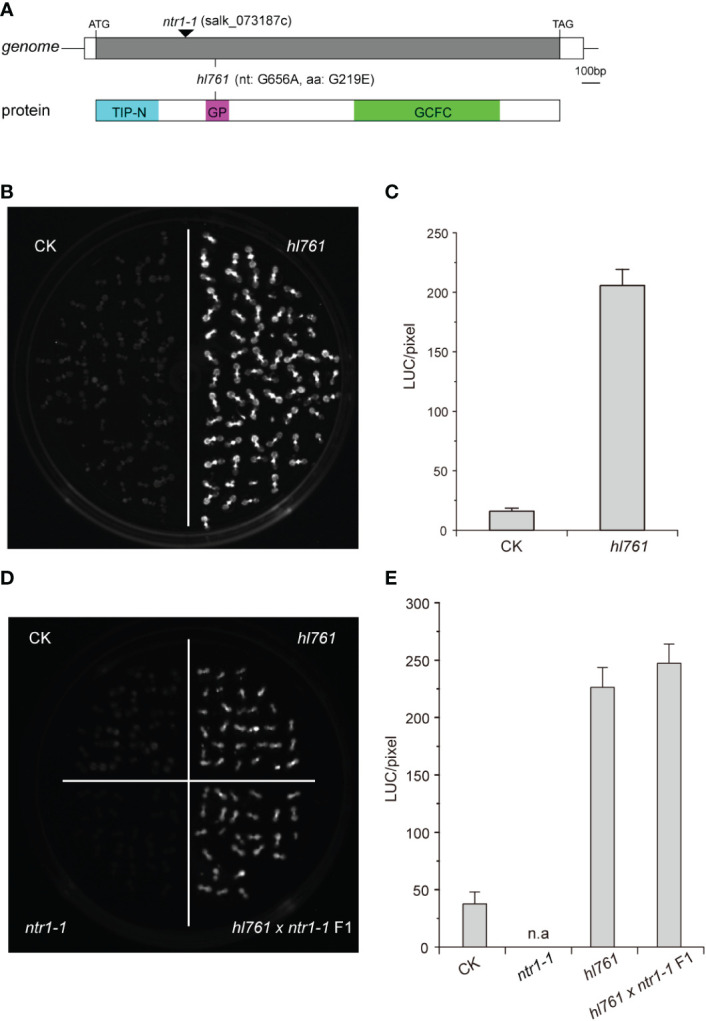
Mutant *hl761* harbors a point mutation of *NTR1*. **(A)** Schematics of the position of *hl761* mutations in the genome and protein. The black triangle shows the insertion place for T-DNA mutant *ntr1-1*. The color boxes in the lower panel indicate the conserved domains of NTR1. TIP-N, Tuftelin interacting protein N terminal domain; GP, G-patch domain; GCFC, GC-rich sequence DNA-binding factor-like protein domain. **(B)** LUC activity in 38°C 1-h heat-treated *hl761* mutant and control plant (CK) seedlings. **(C)** Quantification of LUC activities in seedlings shown in **(B)** Error bar stands for SE, n = 20. **(D)** LUC activity for the cross-complementation test. Samples were 38°C heat treated for 1 h. **(E)** Quantification of LUC activities in seedlings shown in **(D)** Error bar stands for SE, n = 10. n.a, not applicable; CK, background line for *hl761.*.

**Figure 2:**
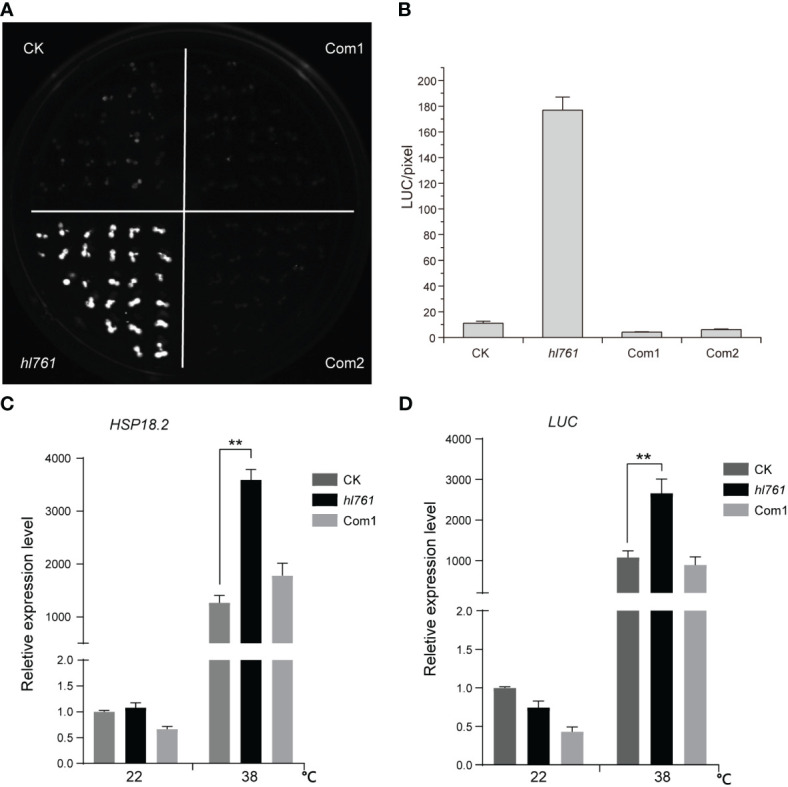
Heat-induced high LUC activity in *hl761* is complemented by introduced *NTR1*. **(A)** LUC activity in 38°C 1-h heat-treated seedlings of control plant (CK), *hl761* mutant, and two independent complemental reference lines, which have single copied *pNTR1:NTR1* transgene. **(B)** Quantification of LUC activities in seedlings shown in **(A)** Error bar stands for SE, n = 20. **(C)** Relative expression of native *HSP18.2* in 38°C 1-h heat-treated seedlings of CK, *hl761*, and complemental line 1 (Com1). The expression is relative to that of CK without heat treatment. Error bars indicate the SD of three biological replicates. **(D)** *LUC* gene expression in 38°C 1-h heat-treated seedlings of CK, *hl761*, and complemental line 1 (Com1). The expression is relative to that of CK without heat treatment. Error bars indicate the SD of three biological replicates. Asterisks indicate statistically significant differences (**p < 0.01 in Student’s t-test). CK, background line for *hl761*.

To seek key regulators for HSR, we constructed an EMS mutant pool with plants harboring the reporter gene firefly luciferase (LUC) driven by the promoter the *Arabidopsis HSP18.2*, which is fast and highly induced by HS (Takahashi and Komeda, 1989; Matsuhara et al., 2000), and screening for mutants with altered LUC activity. One mutant, *hl761* (hl for high light), exhibited obviously increased LUC activity compared to the control plants after 1-h heat treatment at 38°C (**Figures 1B, C**). Through mapping, a point mutation localized at gene *NTR1* (*AT1G17070*) was identified, which causes a G to A transition at the 656th nucleotide, in turn resulting in the 219th amino acid change from glycine to glutamate (G219E) (**Figure 1A**). NTR1 protein contains three conserved domains: a Tuftelin interacting protein N terminal (TIP-N) domain at N-terminal, a G-patch domain (GP) at the middle, and a GC-rich sequence DNA-binding factor-like protein (GCFC) domain at C-terminal. The position of *hl761* mutation localized in the GP domain, which has seven highly conserved glycine residues and appears in many RNA binding proteins for an RNA binding function (**Figure S1**). To confirm that the increasing LUC activity is due to *hl761* mutation, we crossed *hl761* to a reported *NTR1* T-DNA insertion allele *ntr1-1* (salk_073187c) (Dolata et al., 2015) and then scored the LUC activity after 38°C heat treatment. As expected, the F1 plants of *hl761* cross *ntr1-1* showed a similar extent increase of LUC activity (**Figures 1D, E**). We also made another complementation test by introducing *pNTR1:NTR1* to *hl761* mutants. The results showed that NTR1 could fully restore the LUC phenotype, as both two independent reference transgenic lines behaved similarly to the control plants for the LUC activity (**Figures 2A, B**). The expression level of gene *LUC* and native *HSP18.2* was also quantified. Although both *LUC* and *HSP18.2* were highly induced by HS, the expression level was much higher in *hl761* mutants than in the control plants (**Figures 2C, D**), which is consistent with the *LUC* protein activity. Together, these results suggest that *NTR1* mutation accounts for the acute increase of LUC in *hl761* mutants.

### *NTR1* is essential for plant HS tolerance

**Figure 3:**
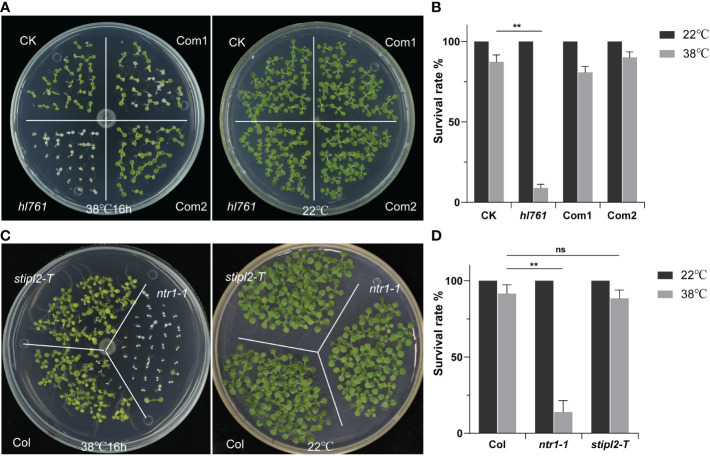
*NTR1* is required for plant heat stress tolerance. **(A)** Heat stress phenotype of CK, *hl761*, and two complemental lines. Left, seedlings were heat treated at 38°C for 16 h and then recovered at 22°C. Right, the same time duration of seedlings is kept at 22°C. CK, background line for *hl761*. **(B)** The survival rate of heat-treated CK, *hl761*, and two complemental lines. Error bars indicate the SD of three biological replicates. Asterisks indicate statistically significant differences (**p < 0.01 in Student’s t-test). **(C)** Heat stress phenotype of T-DNA insertion mutants of *NTR1* and its homolog *STIPL2*. Left, seedlings were heat treated at 38°C for 16 h and then recovered at 22°C. Right, the same time duration of seedlings is kept at 22°C. **(D)** The survival rate of heat-treated Col and T-DNA insertion mutants of *NTR1* and *STIPL2*. Error bars indicate the SD of three biological replicates. Asterisks indicate statistically significant differences (ns, no significance; **p < 0.01 in Student’s t-test).

Next, we speculated whether NTR1 has a function on plant HSR, so we scored the HSR phenotype of *hl761*. Not like the control plants, *hl761* mutants could not recover from 16 h of continuous heat treatment at 38°C, while the complemental lines survived almost the same as the control (**Figures 3A, B**). Furthermore, *ntr1-1*, the null T-DNA insertion allele, was also sensitive to HS treatment (**Figures 3C, D** and **Figure S2**), indicating that NTR1 is vital for plants in HS tolerance. *STIPL2* is the homolog of *NTR1*, with 61.27% similarity between these two proteins. High conservation lies in the G-path domain and the C-terminal GCFC domain but not the N-terminal TIP-N domain between these two proteins (**Figure S1**). Intriguingly, attenuation of *STIPL2* alone did not lead to a decrease in heat tolerance since the null T-DNA mutant of *STIPL2* behaved the same as the wild-type plants (**Figures 3C, D** and **Figure S2**). Coincidentally, although the G-path domain showed high conservation between NTR1 and STIPL2, an arginine in STIPL2 replaces a glycine in NTR1 in the glycine-rich conserved core region (**Figure S1**), which may account for the function variation on heat stress regulation. However, we could not rule out the possible function of *STIPL2* since the wild-type-like phenotype could be due to functional redundancy between *NTR1* and *STIPL2*, which is also implied by the fact that no double mutant of *ntr1-1 stipl2-T* or the *hl761 stipl2-T* could be obtained, as the double mutants may be embryo lethal and could not set seed properly.

In addition to being sensitive to HS, *hl761* mutant plants showed phenotypes such as small plant size, small and ground leaves, and short root length, which was similar to the T-DNA allele *ntr1-1* (**Figures S3A–D**). The expression pattern of *NTR1* was constitutive in plants, as it could be detected in all plant parts (**Figure S3E**).

### *NTR1* mutation results in HSR gene mis-expression under HS

**Figure 4:**
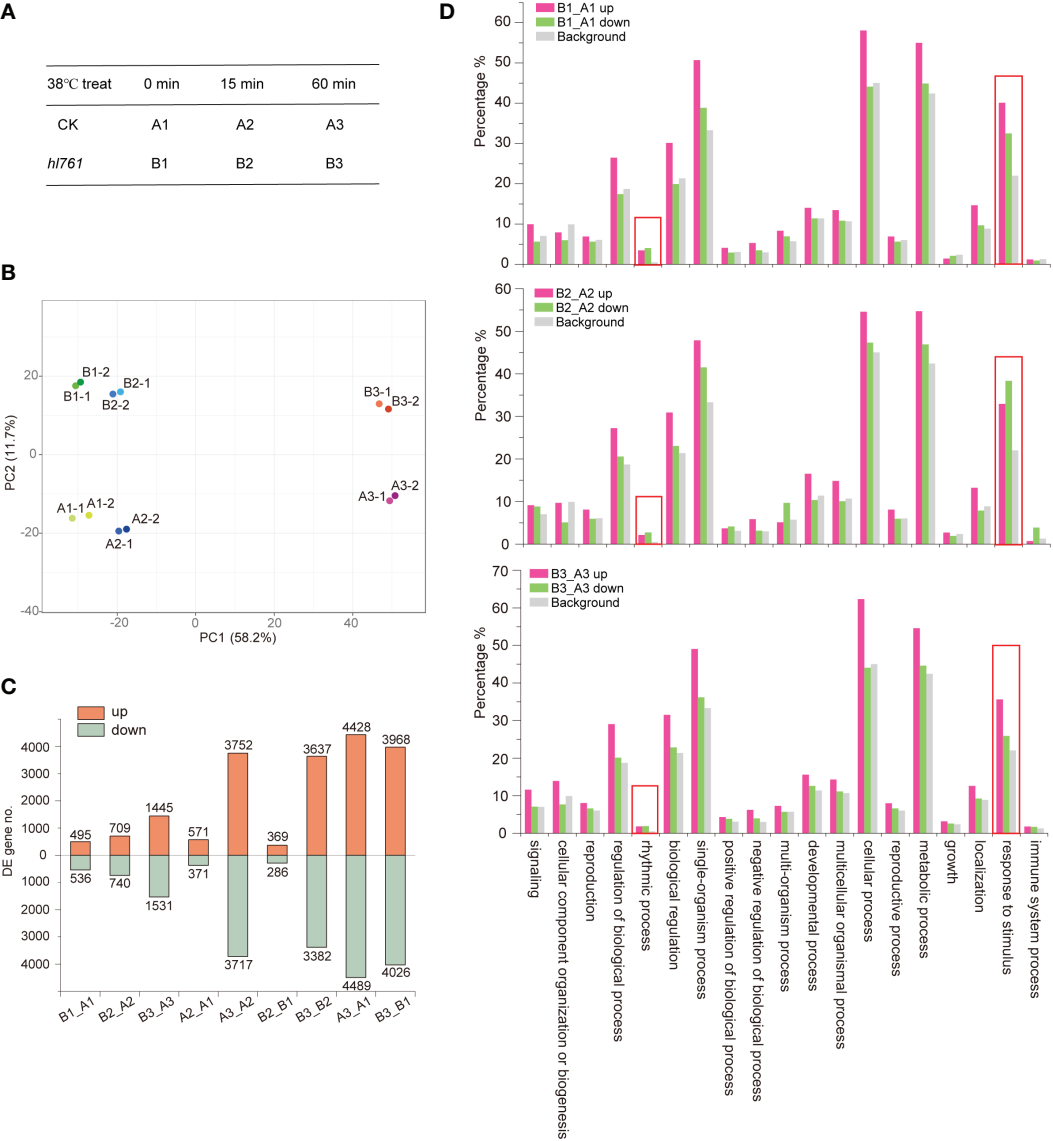
Heat stress triggers gene expression changes in plants. **(A)** The heat stress experiment design for RNA-seq. **(B)** Principal component analysis (PCA) of samples for sequencing. **(C)** Differentially expressed gene (DEG) numbers between different samples. Boxes up the 0 axis of y value represent upregulated DEGs, and boxes below the 0 axis represent downregulated DEGs. The number of DEGs is indicated outside the boxes. **(D)** Gene Ontology (GO) annotation analysis for the biological function of DEGs of *hl761* vs. CK (B_A) at three time points of heat treatment. CK, background line for *hl761.* The y-axis represents the gene fraction of the GO term.

To investigate the mechanism of HS tolerance regulation by *NTR1*, especially at which stage of HSR *NTR1* may influence, we performed transcriptome sequencing with the control plants (A) and *hl761* (B) mutants at three time points of heat treatment at 38°C: 0, 15, and 60 min (**Figure 4A**; the RNA-seq flowchart is illustrated in **Figure S4**). Through principal component analysis (PCA), all the samples could be discriminated at two dimensions. Apparently, heat treatment is the primary factor, as 60-min heat-treated samples were clearly separated from others (**Figure 4B**), while the distribution of 15 min was quite close to the untreated ones (**Figure 4B**), indicating that plants with 15-min heat treatment just lie in the early stage of HSR. For PC2, plants were discriminated according to plant genotype (**Figure 4B**), suggesting that mutation of *NTR1* leads to the separation. When looking at the DEGs, 15-min treatment caused a small portion of genes altering expression in both genetic backgrounds (571 upregulated and 371 downregulated for the control and 369 upregulated and 286 downregulated for *hl761*) (**Figure 4C**, **Table S1**); however, much more gene expression altered for 60 min (3,752 upregulated and 3,717 downregulated for the control and 3,637 upregulated and 3,382 downregulated for *hl761* compared to time point 15 min) (**Figure 4C**; **Table S1**), suggesting that 60-min heat treatment is enough to cause enormous response for gene expression in plants. While comparing *hl761* and the control plants, HS also increased the DEG number (495 upregulated and 536 downregulated for untreated, 709 upregulated and 740 downregulated for 15-min treatment, and 1,445 upregulated and 1,531 downregulated for 60-min time point) (**Figure 4C**; **Table S1**). Considering the number of upregulated and downregulated genes, heat treatment resulted in a comparable scale of altered genes of both different times of treatment and different genetic backgrounds (**Figure 4C**).

For a better understanding of how *NTR1* regulates plant heat tolerance, we performed GO annotation analysis for the biological function of DEGs. As expected, when comparing different time points of heat treatment, for both genotypes, the terms of response to temperature stimulus, response to heat, and heat acclimation were significantly enriched for heat-induced genes (A2_A1 up, A3_A2 up, B2_B1 up, and B3_B2 up) (**Table 1**). However, this was not the case for heat-repressed genes. It is quite intriguing that the term response to cold was also enriched for heat-induced genes (**Table 1**), indicating these genes may respond to both cold and heat. When comparing DEGs between the two genotypes, the GO term of the rhythmic process was significantly enriched for all three time points (B1_A1, B2_A2, and B3_A3) (**Figure 4D**), which is in agreement with the role of *NTR1* in circadian rhythm regulation. Another enriched term in all three time points was response to temperature stimulus (**Figure 4D**), indicating that mutation of *NTR1* results in the expression variation of stimulus-response genes, which is understandable, as decreased heat tolerance was observed in *hl761* mutants. Moreover, significant enrichment for the term of response to heat and response to temperature stimulus was observed for all three time points, especially for 60-min heat treatment with both directions of gene expression (upregulated and downregulated) (**Table 1**), suggesting that HS could significantly cause the mis-expression of HSR genes in *hl761* mutants.

**Table 1:** Genes of response to temperature stimulus and response to heat are enriched in differentially expressed genes (DEGs) between different times of heat treatment or different genotypes.

	Term names	Response to temperature stimulus	Response to heat	Heat acclimation	Response to cold	Cold acclimation
A2_A1 up (571)	Query item Enrichment fold FDR	96 8.64 2.5E−52	69 17.44 9.9E−54	15 16.40 3.5E−11	35 4.58 3.9E−11	5 5.47 0.043
A2_A1 down (371)	Query item Enrichment fold FDR	6 0.82 1	NA NA NA	NA NA NA	5 1.00 1	NA NA NA
A3_A2 up (3752)	Query item Enrichment fold FDR	199 2.75 2.5E−26	108 4.19 2.5E−24	28 4.70 5.8E−07	100 2.01 5.2E−07	15 2.52 0.1
A3_A2 down (3717)	Query item Enrichment fold FDR	64 0.89 1	15 0.58 1	NA NA NA	52 1.05 1	NA NA NA
B2_B1 up (369)	Query item Enrichment fold FDR	82 11.56 4.1E−55	63 24.94 5.5E−59	15 25.69 1.1E−13	25 5.13 4.6E−09	NA NA NA
B2_B1 down (286)	Query item Enrichment fold FDR	5 0.89 1	NA NA NA	NA NA NA	5 1.30 1	NA NA NA
B3_B2 up (3638)	Query item Enrichment fold FDR	209 2.99 2.4E−32	110 4.42 7.4E−27	29 5.04 6.1E−08	112 2.33 2.8E−11	17 2.95 0.014
B3_B2 down (3382)	Query item Enrichment fold FDR	56 0.85 1	11 0.47 1	NA NA NA	46 1.02 1	NA NA NA
B1_A1 up (495)	Query item Enrichment fold FDR	31 3.20 5.70E−06	15 4.35 3.1E−04	NA NA NA	18 2.71 6.8E−03	NA NA NA
B1_A1 down (536)	Query item Enrichment fold FDR	40 3.93 9.6E−10	9 2.48 0.47	NA NA NA	35 5.00 6.4E−11	11 13.12 1.0E-06
B2_A2 up (709)	Query item Enrichment fold FDR	25 1.81 0.088	8 1.62 1	NA NA NA	17 1.79 0.28	NA NA NA
B2_A2 down (740)	Query item Enrichment fold FDR	55 3.85 1.1E−13	15 2.95 0.015	8 6.80 0.003	46 4.69 4.6E−14	13 11.06 2.5E-07
B3_A3 up (1145)	Query item Enrichment fold FDR	59 2.12 2.3E−05	24 2.42 0.006	9 3.92 0.029	41 2.14 7.7E−04	NA NA NA
B3_A3 down (1531)	Query item Enrichment fold FDR	78 2.62 2.7E−09	27 2.55 6.5E−03	10 4.08 0.051	55 2.69 7.8E−07	11 4.49 0.018

Numbers in the parentheses are the no. of DEGs.

NA, not applicable; FDR, false discovery rate.

**Figure 5:**
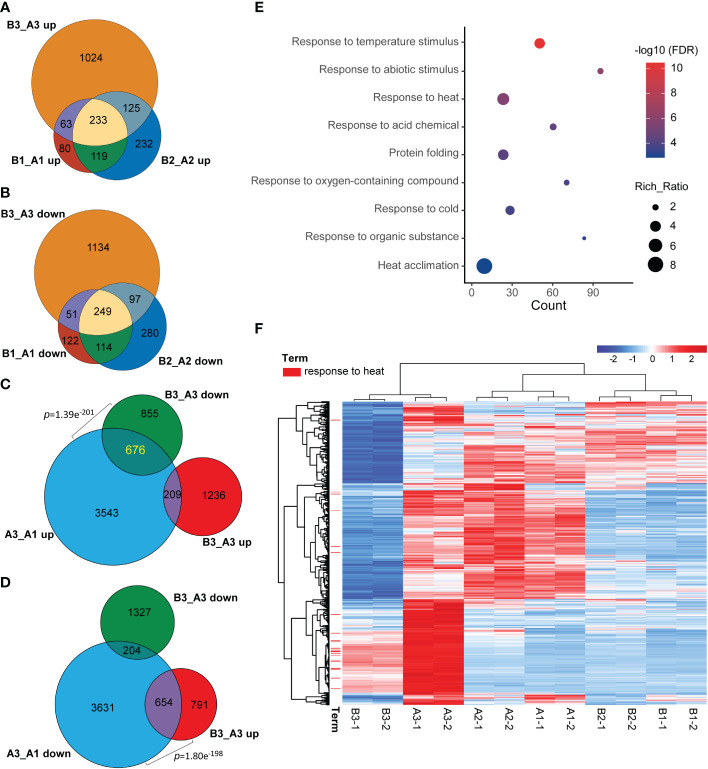
NTR1 promotes HSR gene transcription under heat stress. **(A, B)** Venn diagrams of common genes between upregulated **(A)** and downregulated **(B)** DEGs in *hl761* vs. CK for different times of heat treatment. CK, background line for *hl761.* **(C)** Venn diagram showing the significant overlap between heat stress-induced and downregulated DEGs in *hl761* vs. CK upon 38°C 60-min treatment. **(D)** Venn diagram showing the significant overlap between heat stress-repressed and upregulated DEGs in *hl761* vs. CK upon 38°C 60-min treatment. p-Values (Fisher’s exact test) for overlapping between gene sets are labeled. **(E)** Gene Ontology (GO) annotation analysis for the biological function of the 676 common genes shown in **(C). (F)** Expression heatmap of the 676 common genes shown in **(C)** The heatmap shows the Z-score value of the FPKM of each gene. HSR, heat stress response; DEGs, differentially expressed genes; FPKM, fragments per kilobase of exon per million mapped fragments.

Meanwhile, when comparing common DEGs of *hl761* vs. the control for different times of heat treatment, a great majority of common DEGs were shared between 0- and 15-min heat treatment for both the upregulated and downregulated genes (B1_A1 and B2_A2) (**Figures 5A, B**), and 60-min heat treatment conspicuously amplified the number of DEGs (B3_A3) (**Figures 5A, B**). However, when comparing the DEGs of *hl761* vs. the control at 60-min heat treatment (B3_A3) with heat-inducing (A3_A1 up) or heat-repressing genes (A3_A1 down), significant overlap was observed between downregulated genes in *hl761* (almost a half, 676 out of 1,531) and the heat-inducing ones (**Figure 5C**), and also between upregulated genes in *hl761* (654 out of 1,445) and the heat-repressing ones (**Figure 5D**). Closely checking these common DEGs, among the 676 heat-induced but less expressed in 60-min heat-treated *hl761*, the GO terms of response to temperature stimulus and response to heat were the most two significantly enriched ones (**Figure 5E**). The expression of genes for term response to heat was sharply induced by 60-min heat treatment but significantly less expressed in *hl761* mutants compared to the control plants (**Figure 5F**). However, for genes heat-repressed and upregulated in 60-min heat-treated *hl761*, no significant enrichment of these temperature-related terms was found. Therefore, those heat-induced but downregulated in *hl761* HSR genes may account for the low heat tolerance for *hl761* mutants.

However, if comparing the DEGs for different heat treatment times in the same background (A2_A1, A3_A2, B2_B1, and B3_B2), for both heat-induced and heat-repressed genes, DEGs in *hl761* exceedingly overlapped with those in the control (**Figures S5A, B**). Thus, *hl761* shared a common set of genes in response to HS with the control plants at the gene expression level; in other words, disruption of *NTR1* does not affect the ability to respond to HS for plants.

### A large portion of *HSF*s and *HSP*s are less induced by heat in *hl761* mutants

**Figure 6:**
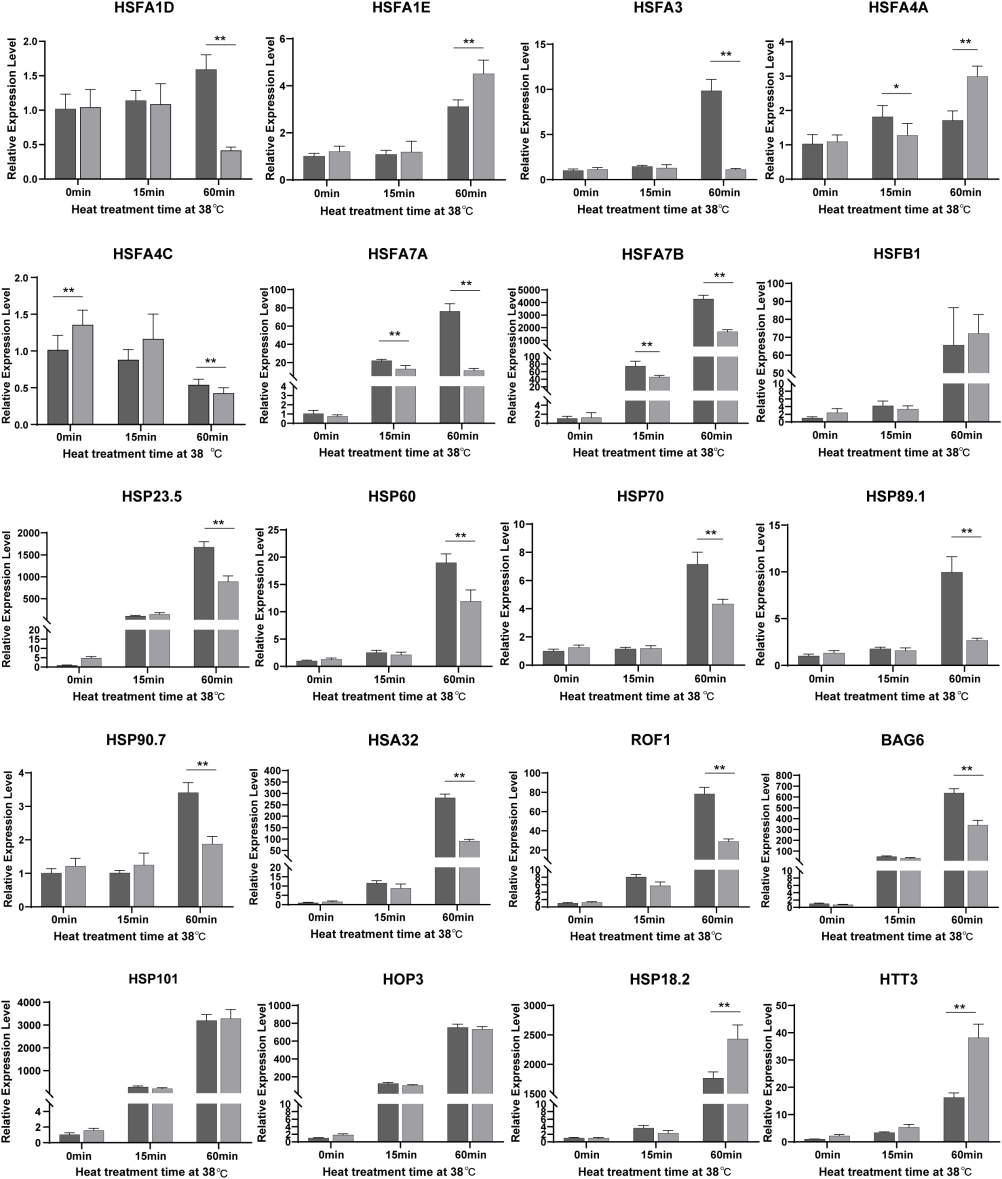
A large portion of *HSF*s and *HSP*s are downregulated by *NTR1* mutation under heat stress. Gene expression in seedlings of CK and *hl761* before heat treatment, and 15 min and 60 min upon 38°C heat treatment were quantified. The expression is relative to that of CK before heat treatment. Error bars indicate the SD of three biological replicates. Asterisks indicate statistically significant differences (*p < 0.05, **p < 0.01 in Student’s t-test). CK, background line for *hl761.*.

To further explore the function of NTR1 in HSR gene regulation during HS and also to verify the RNA-seq data, we quantified the expression of HSFs and HSPs by real-time PCR. Out of 20 genes (**Figure 6**) checked for the same treatment as the RNA-seq samples, 19 showed a similar tendency of variations between both the two genotypes and different treatment times. All the genes checked were induced by heat, especially by 60-min treatment except *HSFA4C*. The majority of them were less induced in *hl761* mutants compared to the control upon 60-min heat treatment, such as *HSFA1D*, *HSFA3*, *HSFA7A*, *HSFA7B*, *HSFA1B*, *HSP23.5*, *HSP60*, *HSP70*, *HSP89.1*, *HSP90.7*, *HSA32* (*HEAT STRESS ASSOCIATED 32*), *ROF1*, and *BAG6* (*BCL-2-ASSOCIATED ATHANOGENE 6*) (**Figure 6** and **Figure S6**). However, a few genes such as *HSFA1E*, *DREB2A*, *HSP18.2*, *HTT1*, *HTT2*, and *HTT3* showed more expression in *hl761* for 60-min heat treatment (**Figure 6** and **Figure S6**). Others such as *HSFB1*, *HSFB2A*, *HSFB2B*, *HSFA2*, *HSP101*, *HOP3*, and *APX1* had no obvious difference (**Figure 6** and **Figure S6**). At the early stage (15-min treatment) of HS, *HSFA7A* and *HSFA7B* were strongly induced by heat in both backgrounds but were much less expressed in *hl761* mutants (**Figure 6**). Consistent with the RNA-seq results, these data suggested that the integrative effects of the mis-expression for HSR genes, especially those less induced in *hl761*, result in the decreased HS tolerance of the mutants.

### *NTR1* mutation amplifies the mis-splicing of HSR genes induced by HS

**Figure 7:**
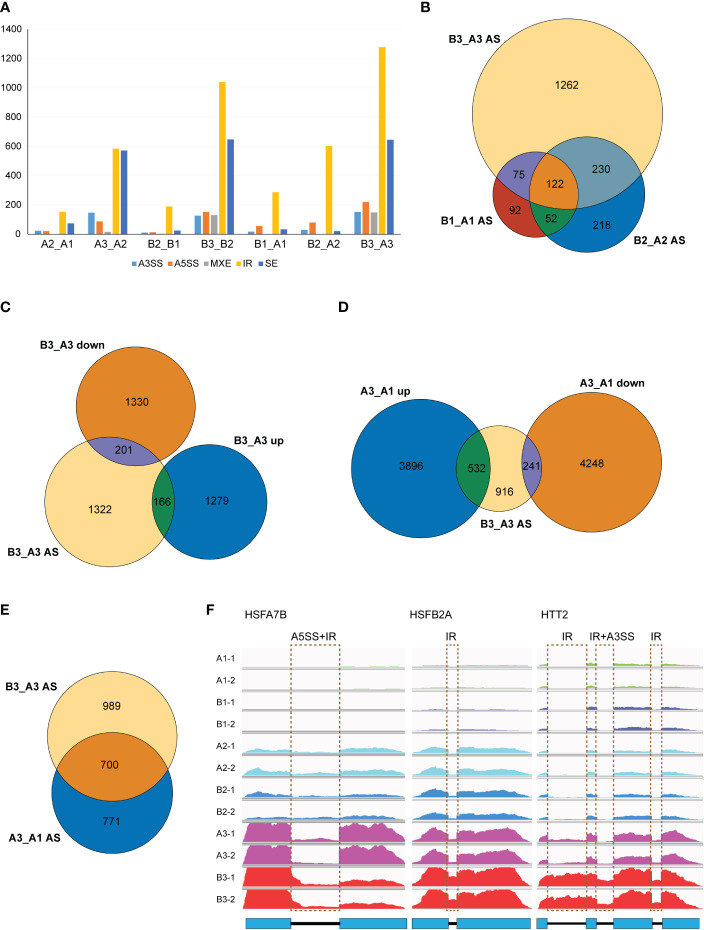
*NTR1* mutation amplifies the alternative splice of HSR genes induced by heat stress. **(A)** Differential AS events compared between plants with different times of heat treatment or between different genotypes. A3SS, alternative 3′-splice site; A5SS, alternative 5′-splice site; MXE, mutually exclusive exon; IR, intron retention; SE, skipped exon. **(B)** Venn diagrams of common genes with different AS events between *hl761* mutants and CK at different times of heat treatment. **(C)** Venn diagrams of common genes with different AS events and DEGs of *hl761* vs. CK after 60-min heat treatment. **(D)** Venn diagrams of common genes between genes with different AS events of *hl761* vs. CK after 60-min heat treatment and heat-induced or heat-repressed genes by 60-min heat treatment. **(E)** Venn diagram of common genes between genes with different AS events of hl761 vs. CK after 60-min heat treatment and genes with heat-induced AS events. **(F)** Examples of heat stress response (HSR) genes with altered AS induced by heat stress. CK, background line for *hl761*; AS, alternative splicing; DEGs, differentially expressed genes.

As NTR1 was reported for its essential role in AS regulation, we speculated whether NTR1 is also involved in AS controlling during HS. We analyzed the differential AS between different samples by rMATS. It is clear that heat treatment, especially 60-min heat treatment, promotes AS in plants, with a steep increase of IR and skipped exon (SE) events in both the control and *hl761* mutants (A3_A2 and B3_B2) while much more IR in *hl761* for 60- *vs.* 15-min heat treatment (B3_B2) (**Figure 7A**). When comparing AS events between the two genotypes, many IR events occurred in *hl761* without heat treatment (B1_A1) (**Figure 7A**), consistent with the previous report (Ling et al., 2018). The 60-min heat treatment could induce all types of AS events obviously (B3_A3), with much more IR and SE events (**Figure 7A**).

**Figure 8:**
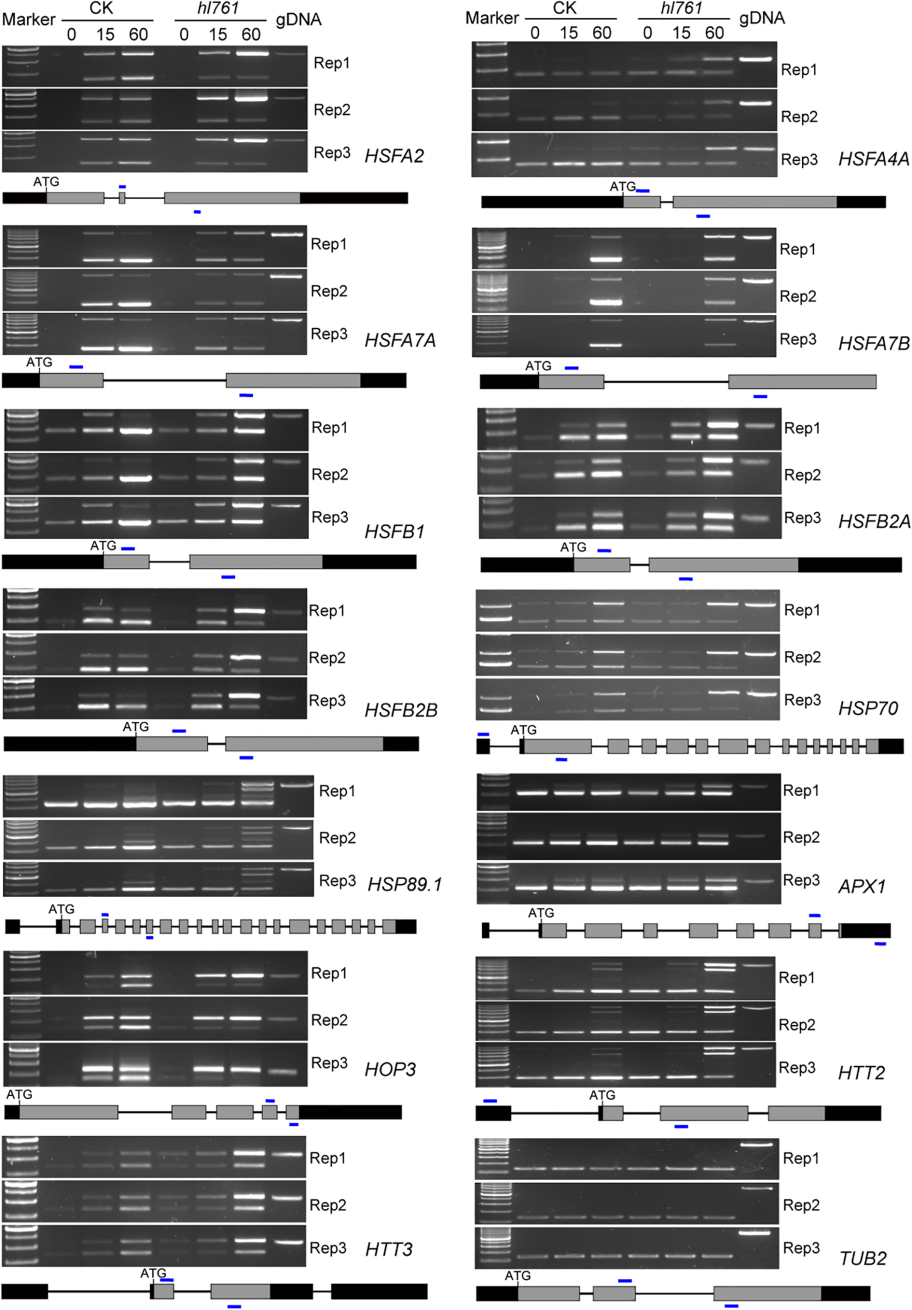
*NTR1* mutation alters the AS of *HSF*s and *HSP*s under heat stress. Three biological repeats are presented. Below the gel pictures are the schematic of the gene structure. Gray boxes indicate the exons, black boxes indicate the 5′ or 3′-UTR, lines in between indicate the introns, and blue lines show the primer position for RT-PCR. CK, background line for *hl761*; AS, alternative splicing.

Considering genes with differential AS, like the number of DEGs, heat treatment significantly exaggerated the number of differentially spliced genes (341 for B1_A1, 622 for B2_A2, and 1,689 for B3_A3) (**Figures 7B, F**). GO analysis of biological process with these AS genes in *hl761* under 60-min heat treatment (B3_A3) showed that in addition to terms related to RNA metabolic process, the terms response to temperature stimulus and response to heat were significantly enriched (**Figure S7**). We also examined the AS events for several HSR genes by RT-PCR. Most genes examined showed more IR in *hl761* than the control after 60-min heat treatment, such as *HSFA2*, *HSFA4A*, *HSFA7A*, *HSFA7B*, *HSFB1*, *HSFB2A*, and *HSFB2B* (**Figure 8**), although some of them (*HSFA2*, *HSFB1*, *HSFB2A*, *HSFB2B*, *HOP3*, *APX1*) showed no obvious difference of expression in *hl761* or even higher in *hl761* after 60-min heat treatment (*HSFA4A*, *HTT2*, and *HTT3*).

Thus, it is interesting to know the connection between AS and gene transcription. We examined common genes between differentially spiced (B3_A3 AS) and DEGs of 60-min heat-treated *hl761* vs. the control (B3_A3 up and B3_A3 down). Surprisingly, only a small portion of AS genes were differentially expressed (about 20%), and no noticeable bias toward NTR1 promoted or suppressed genes (**Figure 7C**). The transcription for the majority of AS genes (1,322 out of 1,689) was similar between both backgrounds (**Figure 7C**). However, the expression of about one-half of B3_A3 AS genes was heat responsive, more than threefold for heat-induced (532 of 1,689) to heat-repressed genes (166 of 1,689) (**Figure 7D**). Moreover, a high overlap was observed for heat-induced AS (A3_A1 AS) and NTR1-dependent AS (B3_A3 AS) (**Figure 7E**), indicating that during HS, mutation of *NTR1* may result in similar AS events to heat-induced ones. All these data suggested that NTR1 not only affects gene transcription but also functions during HS for maintaining normal splicing of HSR genes.

### NTR1 shows a preference for impeding the expression of intron-containing genes

**Figure 9:**
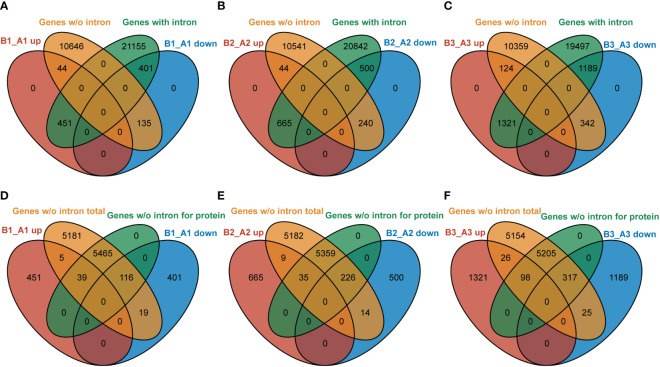
NTR1 shows a preference for impeding the expression of intron-containing genes. **(A–C)** Venn diagrams of common genes between DEGs of *hl761* mutants *vs.* the control plants (CK) before heat treatment **(A)**, and 15-min **(B)** and 60-min **(C)** heat treatment and intron-containing genes or that without intron. **(D–F)** Venn diagrams of common genes between DEGs of *hl761* mutants *vs.* the control plants (CK) before heat treatment **(D)**, and 15-min **(E)** and 60-min **(F)** heat treatment and total genes without intron or subset protein-coding genes. DEGs, differentially expressed genes.

In *Arabidopsis*, about one-third of the genes have no introns (data from gene annotated in TAIR 10). NTR1, as an accessory component of spliceosome, is involved in pre-mRNA splicing. Our data indicated that NTR1 also affects gene transcription, and no clear bias was observed for gene repression or promotion. Then, it is intriguing to ask whether there is any connection between transcription and splicing. We compared the DEGs in *hl761* of discrete time of heat treatment for genes with or without intron. For NTR1-promoted genes (B_A down), more genes with intron were affected than genes without intron (ratio about 3:1 for B1_A1 and B3_A3 downregulated genes) (**Figures 9A–C**). However, for NTR1-repressed genes (B_A up), NTR1 affected far more intron-containing genes (ratio more than 10:1 for all three time points of heat treatment) (**Figures 9A–C**), although the totally similar scales of genes were affected for both directions (upregulated and downregulated). Therefore, NTR1 is preferred to suppress transcription for intron-containing genes, which is understandable as the involvement of NTR1 in the spliceosome disassembly complex. For genes without intron, about half of them are translated to proteins in *Arabidopsis*. Among them, NTR1 had more impact on the expression of protein-coding genes for both promoting and suppressing expression, while much more frequently for the promotion (more than 10:1 for protein-coding to no coding genes) (**Figures 9D–F**) than suppression (ratio about 4:1) (**Figures 9D–F**).

### HS suppresses the expression of *NTR1* and components of the NTR1-associated complex

**Figure 10:**
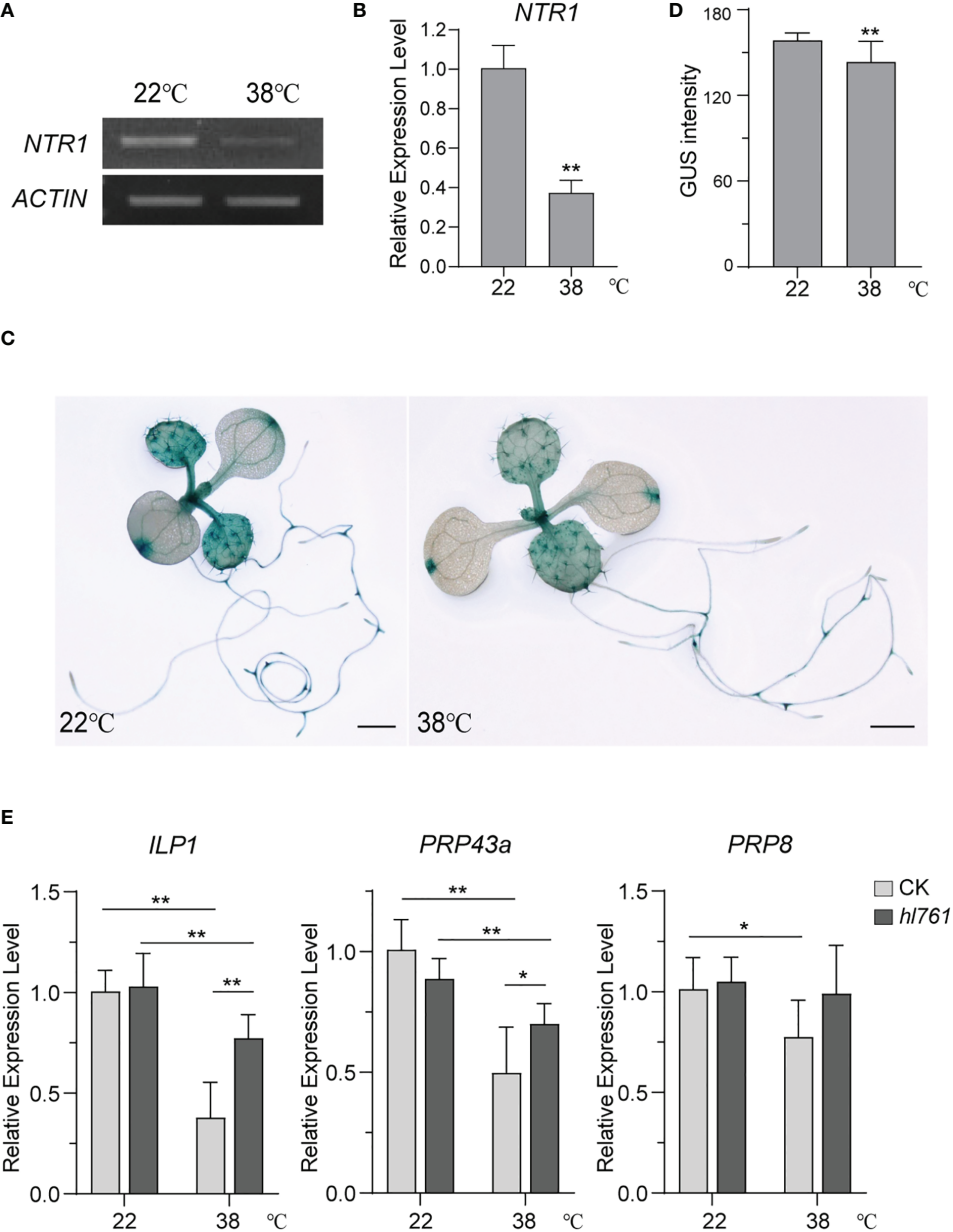
HS suppresses the expression of *NTR1* and other components of the NTR1-associated complex. **(A)** Semi-quantification of the expression of *NTR1* upon 1-h heat treatment at 38°C. **(B)** The expression of *NTR1* upon 1-h heat treatment at 38°C quantified by qPCR. The expression is relative to that of untreated samples. Error bars indicate the SD of three biological replicates. Asterisks indicate statistically significant differences (**p < 0.01 in Student’s t-test). **(C)** β-Glucosidase (GUS) staining of *pNTR1:GUS* transgenic plants with (right) or without (left) 38°C 1-h heat treatment. Bar = 1 mm. **(D)** Quantification of GUS intensity for plants in panel **(C)** Error bar stands for SD, n = 20. Asterisks indicate statistically significant differences (**p < 0.01 in Student’s t-test). **(E)** Relative expression of *ILP1*, *PRP43a*, and *PRP8* in 38°C 1-h heat-treated seedlings of CK and *hl761*. The expression is relative to that of CK without heat treatment. Error bars indicate the SD of three biological replicates. Asterisks indicate statistically significant differences (*p < 0.05, **p < 0.01 in Student’s t-test). CK, background line for *hl761*; HS, heat stress.

*NTR1* is critical to plant HS tolerance and for the regulation of HSR gene expression during HS. We were probing whether the expression of *NTR1* itself is regulated by HS. Then, *NTR1* expression levels after heat treatment were examined. Interestingly, 1-h heat treatment at 38°C could reduce almost two-thirds of *NTR1* expression (**Figures 10A, B**), suggesting that *NTR1* expression is suppressed by HS. To further confirm the repression effect on *NTR1* expression by HS, the transgenic plants harboring the *GUS* reporter gene driven by *NTR1* promoter (*pNTR1:GUS*) were subjected to heat treatment for 1 h at 38°C. As expected, the GUS signals were clearly reduced after heat treatment (**Figures 10C, D**), confirming the suppression of *NTR1* by HS.

Since NTR1 is involved in the complex with ILP1, PRP43, and PRP8 and is closely associated with ILP1, we speculated whether the mutation of other complex components also causes the decrease of plant HS tolerance. Not surprisingly, *ilp1* mutants behaved similarly to *ntr1* mutants with reduced tolerance to HS (**Figure S8**). Therefore, we speculated that HS may also result in the suppression of other components of the NTR1-associated complex. As expected, just like *NTR1*, the expression of *ILP1*, *PRP43a*, and *PRP8* in the control plants was significantly decreased after heat treatment (**Figure 10E**), and this reduction was also detected for *ILP1* and *PRP43a* in the *hl761* background (**Figure 10E**). Furthermore, the expression of these three genes in *hl761* mutants was higher than that in the control plants, although there was no statistical difference for *PRP8*, suggesting that NTR1 impedes the transcription of these genes during HS (**Figure 10E**). Therefore, HS suppresses the expression of the NTR1-associated complex.

## Discussion

Stress induces the reprogramming of the transcriptome in plant cells. Cold treatment leads to a rapid transcriptional and AS activity wave in a few hours (Calixto et al., 2018). HS also quickly evokes gene expression variation and AS events in plants (Ling et al., 2021; Ding and Yang, 2022). Here, we showed that 1-h heat treatment at 38°C is enough to result in transcription changes and AS for thousands of genes. *NTR1*, as an accessory component of spliceosome, plays an important role in this process.

*NTR1* affects the expression of HSR genes at both transcriptional and post-transcriptional levels under HS. The majority of HS-induced genes checked were less induced in *hl761* mutants than in the control upon 60-min heat treatment, and genes for response to temperature stimulus and response to heat were significantly enriched in common genes for heat-induced but downregulated in 60-min heat-treated *hl761*. Moreover, *NTR1* mutation amplified HS induced AS especially IR events and caused different AS (mainly IR) of HSR genes during HS. Thus, the mis-expression and the false splicing of the pre-mRNAs for these HSR genes in *ntr1* mutants may account for the reduced HS tolerance of the mutants. Notably, only a small portion of NTR1-dependent AS genes were differentially expressed in *hl761* after the 60-min heat treatment (**Figure 7C**), indicating that the majority of these genes were regulated only by AS under HS.

*NTR1* was reported for its role in circadian rhythm and miRNA biogenesis regulation (Jones et al., 2012; Wang et al., 2019). It is interesting to know whether NTR1 regulates plant HS response through the regulation of miRNAs. As expected, the miR167 precursor, a potential target of NTR1 (Shen et al., 2014), was detected in the downregulated DEGs in *hl761* before heat treatment (B1_A1 down, **Table S1**). However, we found that only two microRNA precursors, miR824 and miR850, are downregulated in *hl761* after 1-h heat treatment (B3_A3 down, **Table S1**). The function of miR850 has not been documented yet, and miR824 was reported for the regulation of stomatal development and flowering (Kutter et al., 2007; Hu et al., 2014; Szaker et al., 2019). HS elevates the expression of miR824 (Szaker et al., 2019), which is less induced in 1-h heat-treated *hl761* mutants. Therefore, it is possible that NTR1 adjusts plant development through the miR824 pathway during HS, which is worthy of further investigation.

Although researchers have noticed that HS stimulates AS, in plants, the exact mechanism is not well understood. The research for AS regulation in HS normally focused on the AS events for particular key TFs. The splicing factors involved in HS response are mainly related to the SR proteins, whose expression is regulated by AS (Zhao et al., 2021). However, whether HS regulates other splicing-related factors are seldom reported. As a spliceosome accessory, NTR1 is essential for plants in maintaining the correct splice of pre-mRNAs under HS, since much more AS events especially IR events were observed in *hl761* mutants compared to the control plants after HS. Meanwhile, HS strongly reduces the expression of *NTR1* and other components of the NTR1-associated complex. There appears to be feedback presented for the regulation of NTR1 complex during heat response as the expression of *ILP1* and *PRP43a* was much higher in *hl761* mutants after heat treatment. However, how HS suppresses the transcription of *NTR1* and NTR1 complex requires further investigation. Therefore, HS increases the false AS, especially because IR may have resulted from the suppression of the NTR1-associated complex.

At the transcription level, plants could quickly respond to HS. A 15-min heat treatment at 38°C is adequate to trigger expression change of early response HSR genes, for instance, activating expression of a big portion of small *HSP*s and transcription factors such as *MBF1C*, *HSFA2*, *HSFA7A*, and *HSFA7B*. A 60-min heat treatment at 38°C is enough to cause the expression change of thousands of HSR genes, which means a fast and high amount of requirements for the transcription of HS-induced genes. Considering the function of NTR1 on splicing and the co-localization of NTR1 with RNA polymerase II (RNA PolII) (Dolata et al., 2015), it is likely that NTR1 or NTR1-associated spliceosome disassembly complex may have a negative effect as physical obstacles on RNA PolII occupation on chromatins when the fast transcription of heat-induced genes occurs during plant heat responses.

Based on all the data we obtained here, we propose a model for the strategy that plants may adopt to respond to HS. When encountering HS, a huge number of HSR genes especially as the HSFs and the HSPs are induced. On the one hand, the NTR1-associated complex assists the process of pre-mRNAs. On the other hand, great transcriptional events happen to fulfill the high requirement of HSR gene products, which makes the presence of NTR1 or NTR1-associated spliceosome disassembly complex a restrictive factor for transcription. In such a scenario, plants reduce the expression of the NTR1-associated complex to ensure the transcription. Thus, the decrease of NTR1 machinery results in the accumulation of improper spliced products, which eventually cause harm to the plants.

## Data availability statement

The data presented in the study are deposited in the NCBI repository, accession number: PRJNA896322.

## Author contributions

LH, QW, YJ, and YF performed the experiments. YW, WY, and HS conceived and designed the experiments. YW wrote the manuscript. WY revised it. All authors contributed to the article and approved the submitted version.
