# Supplementary material for: NTR1 is involved in heat stress tolerance through mediating expression regulation and alternative splicing of heat stress genes in Arabidopsis
Source: Front Plant Sci. 2023 Jan 10;13:1082511. doi: 10.3389/fpls.2022.1082511 (PMC9871932; doi:10.3389/fpls.2022.1082511)
Supplement: Supplementary file 1 [file DataSheet_1.zip › Supplemental Figures.docx]

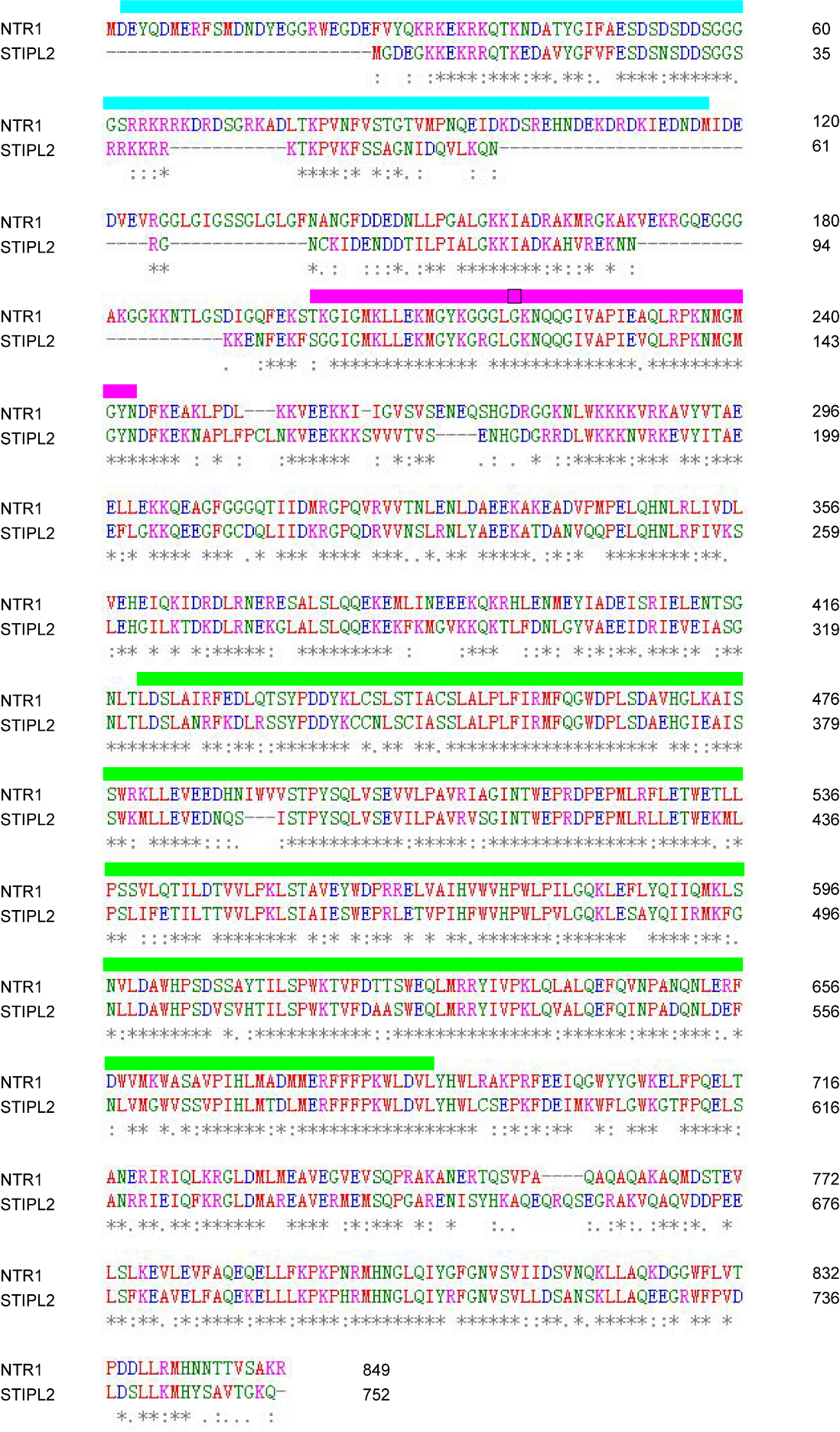


**Fig S1.** Sequence alignment of NTR1 and STIPL2 proteins. The alignment was performed by Clustal Omega. The color boxes up the sequence indicate the conserved protein domains. Cyan, TIP-N domain; pink, G-path domain; green, GCFC domain. The black box in the pink one presents the mutation site of *hl761*.

*
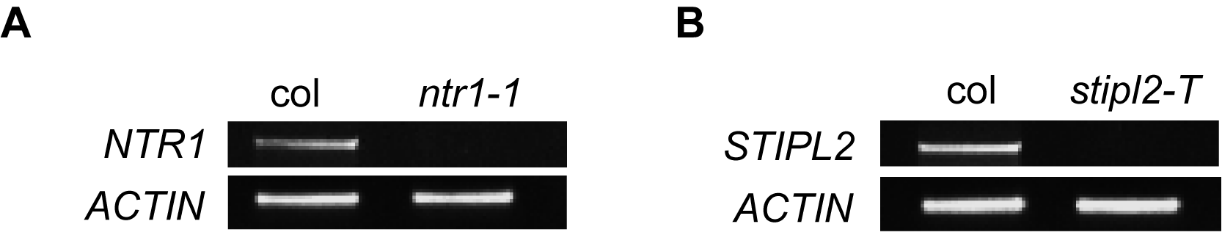
*

**Fig S2.** The T-DNA insertion mutants of *NTR1* and *STIPL2* are null alleles. **A.** and **B.** Semi-quantification of the expression of *NTR1* (**A**) and *STIPL2* (**B**) in the respective T-DNA insertion mutants. *ACTIN* was used as internal control with PCR cycle numbers 23; For *NTR1* or *STIPL2*, the PCR cycle numbers were 35.


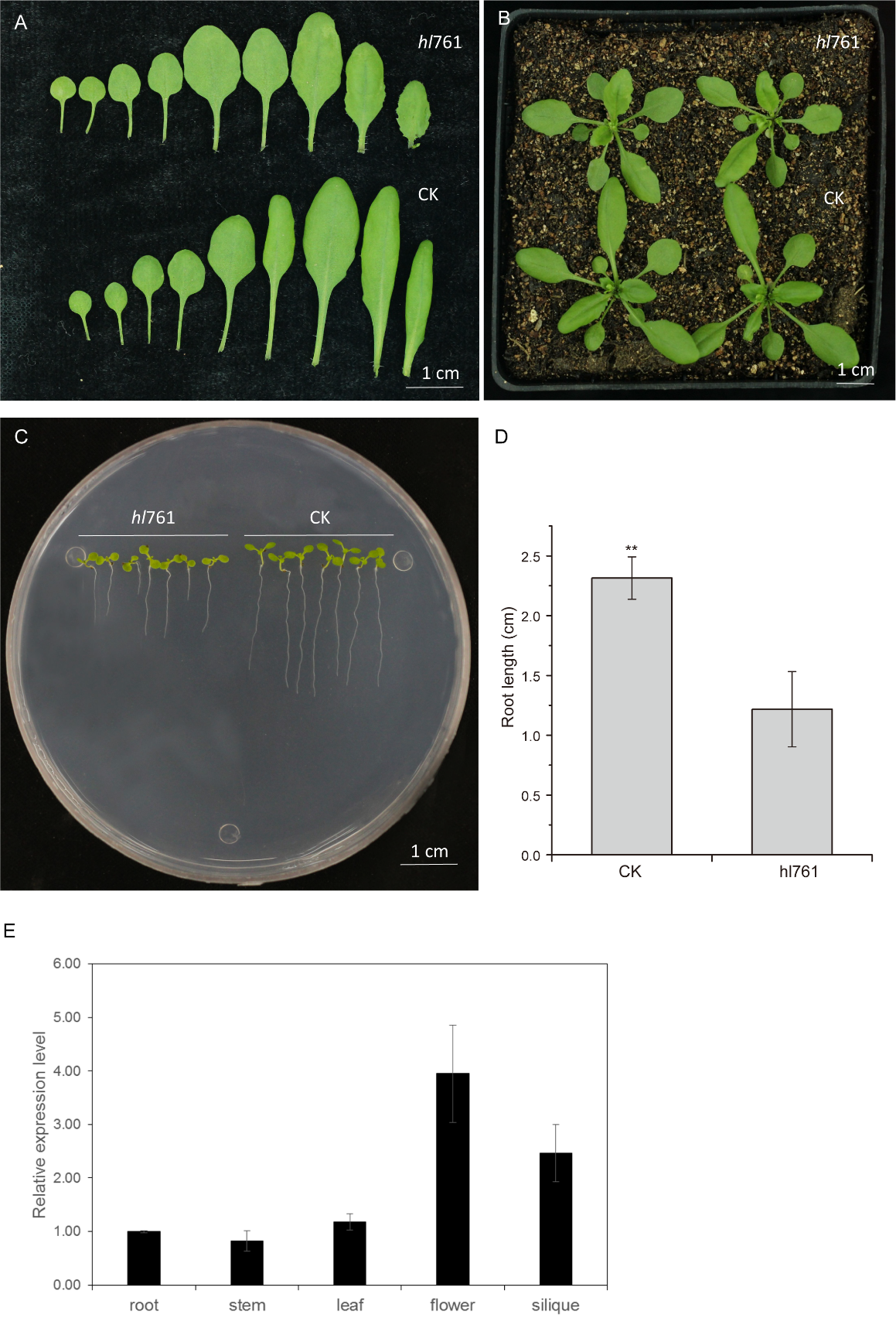


**Fig S3.** The growth phenotypes of *hl761* mutants. **A.** The leaf shape of *hl761* mutants and the control plants. **B.** The plant size of *hl761* mutants and the control plants. Plants around 20 DAG plants in long day (LD) conditions were showed. **C.** The root length of 7 DAG old seedlings of *hl761* mutants and the control plants. **D.** Quantification of the root length of seedlings showed in C. Error bar stands for SD. Asterisks indicate statistically significant differences (*p < 0.05, **p < 0.01 in the Student's t-test). CK, background line for *hl761.* **E.** *NTR1* expression in different plant organs. The expression was relative to that in roots. Error bars indicate the SD of three biological replicates.


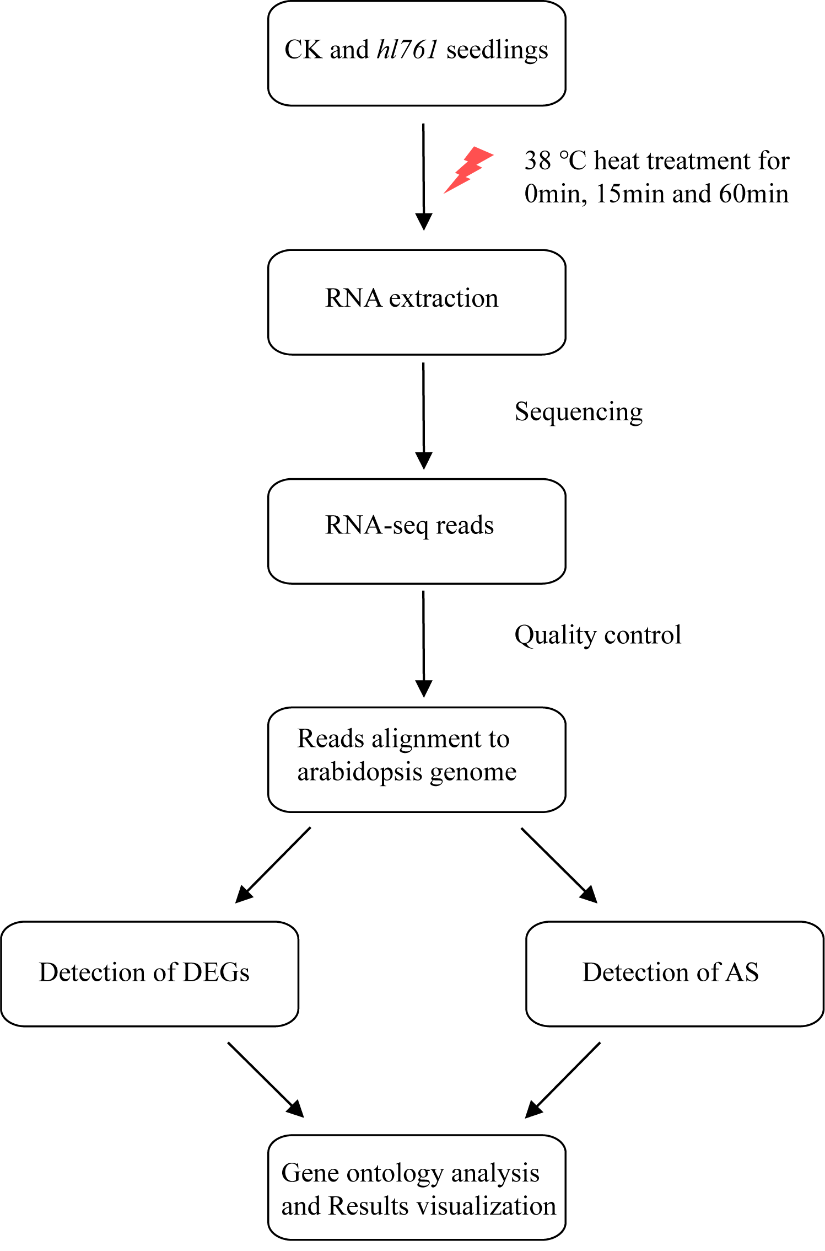


**Fig S4.** The flowchart of RNA-seq methodology. In short, the 10 DAG seedlings of hl761 mutants and the control plants (CK) were heat treated (38℃) and collected at three time points (0 min, 15 min and 60 min). Total RNA was extracted then sequenced by Illumina HiSeq 2500 platform. RNA-seq reads (after Quality control by FASTQC) were aligned to the Arabidopsis reference genome (TAIR10) using HISAT2 and gene counts were determined using HTSeq. The differentially expressed genes (DEGs) were identified with Bioconductor package DESeq2 with a fold change ≥ 1.5 or ≤ 2/3 with an FDR (False Discovery Rate) ≤ 0.05. For differential AS events, analysis was performed with rMATS (with a threshold of 0.05 and false discovery rate ≤ 0.05). Gene ontology (GO) enrichment analysis was analyzed using agriGO.


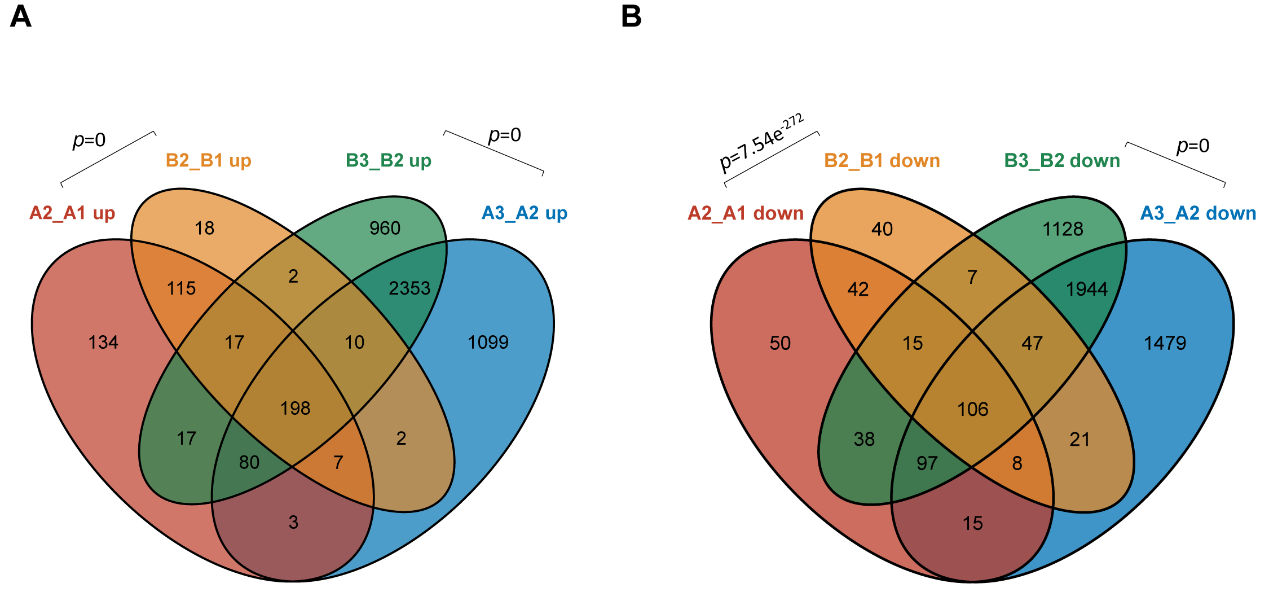


**Fig S5.** Significant DEG overlap for HSR genes in the *hl761* mutants and in the control plants after heat treatment. **A.** Venn diagram showing significant overlap between 38℃ 15 min heat treatment induced genes in *hl761* mutants and that in the control plants, or between prolonged heat treatment induced genes in these two genotypes (38℃ 60 min vs 15 min heat treatment). **B.** Significant overlap between 38℃ 15 min heat treatment repressed genes in *hl761* mutants and that in the control plants, or between prolonged heat treatment repressed genes in these two genotypes (38℃ 60 min vs 15 min heat treatment). P values (Fisher’s exact test) for overlapping between gene sets are labeled. CK, background line for *hl761.*


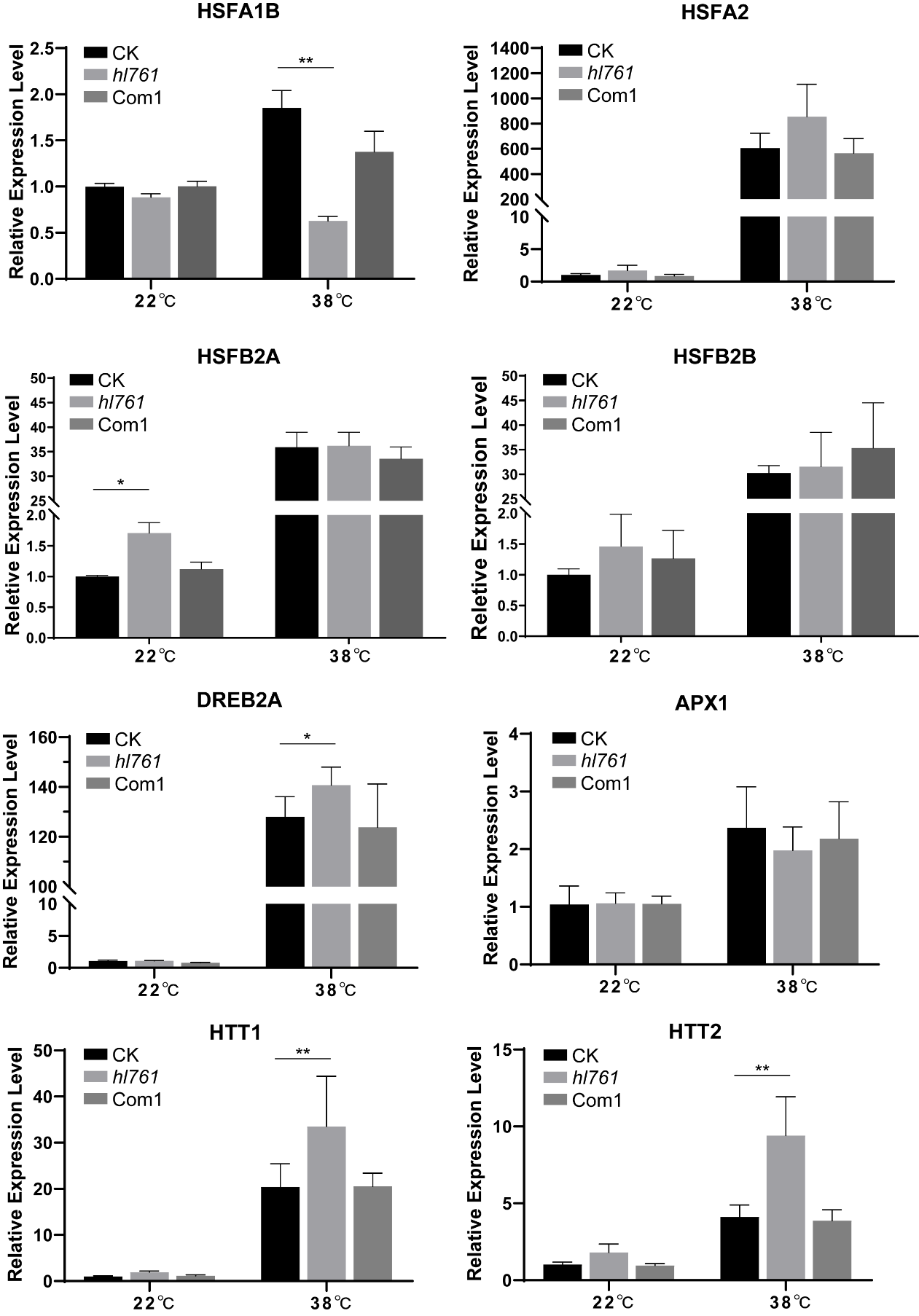


**Fig S6.** Expression of more HSR genes upon heat stress. Gene expression in seedlings of the control plants (CK), *hl761* and the complemental line 1 with or without 38℃ 60 min heat treatment were quantified. The expression is relative to that of CK without heat treatment. Error bars indicate the SD of three biological replicates. Asterisks indicate statistically significant differences (*p < 0.05, **p < 0.01 in the Student's t-test). CK, background line for *hl761.*


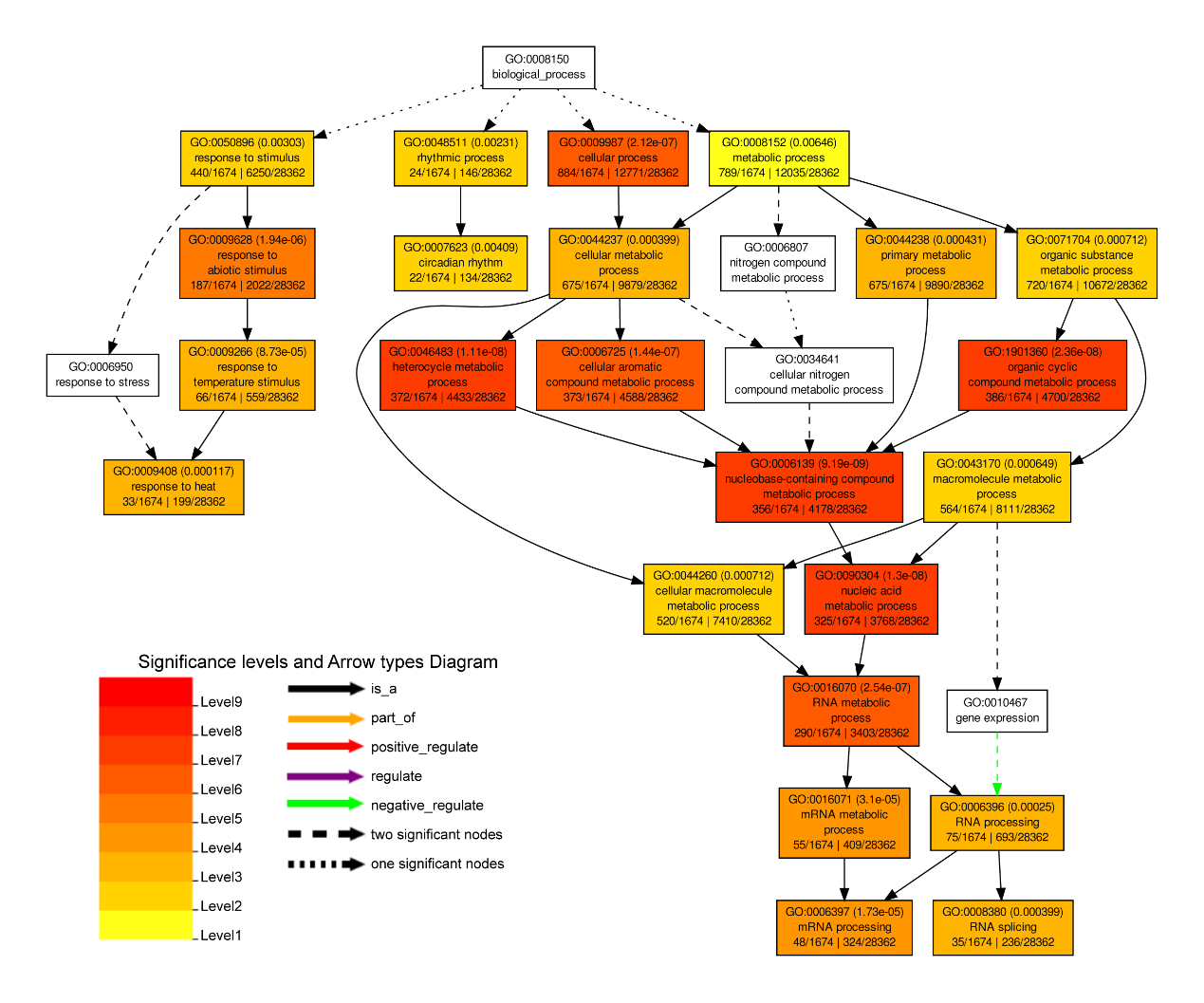


**Fig S7.** Genes of response to heat are enriched in differential alternatively spliced (AS) genes in heat treated *hl761*. Gene Ontology annotation (GO) analysis for the biological function of the differential alternatively spliced (AS) genes in 38℃ 60 min heat treated *hl761* vs heat treated control plants.


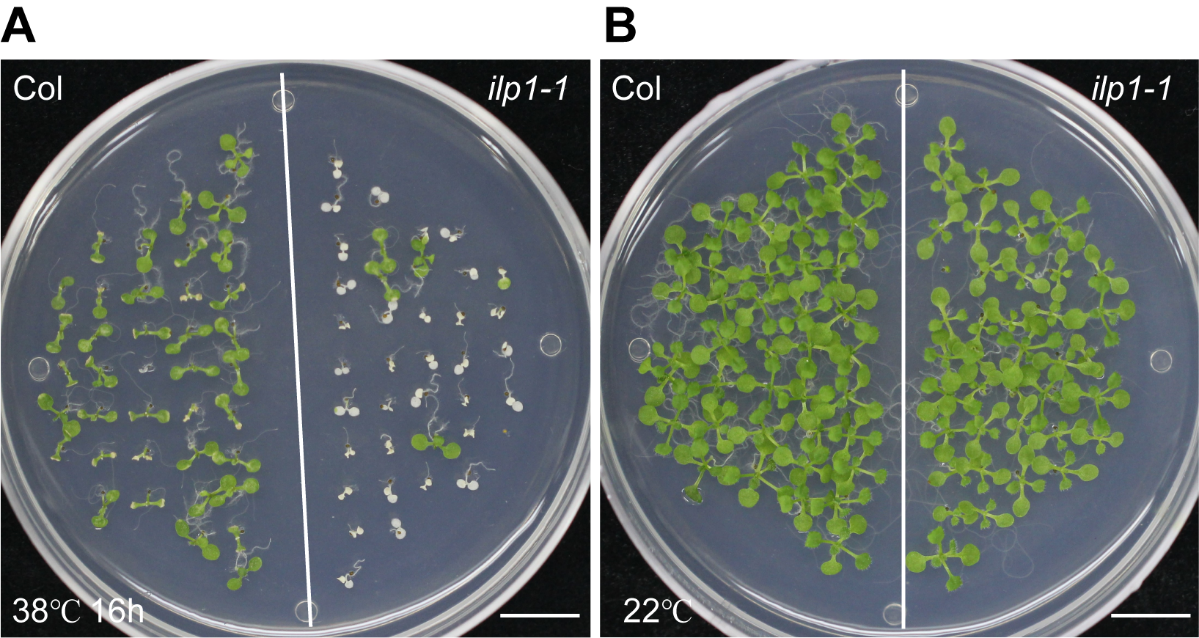


**Fig S8.** Heat stress phenotypes of the *ilp1* T-DNA insertion mutant. **A.** seedlings of the wild-type Col and the T-DNA insertion mutant *ilp1-1* were heat treated at 38℃ for 16 hours then moved to 22℃ to recover for 5 days. **B.** The same time duration of seedlings kept at 22℃.
