# Supplementary material for: NTR1 is involved in heat stress tolerance through mediating expression regulation and alternative splicing of heat stress genes in Arabidopsis
Source: Front Plant Sci. 2023 Jan 10;13:1082511. doi: 10.3389/fpls.2022.1082511 (PMC9871932; doi:10.3389/fpls.2022.1082511)
Supplement: Supplementary file 1 [file DataSheet_1.zip › Table S2. Primers used in this paper.doc]

| **Table S2.** Primers used in this paper | |  |  |
| --- | --- | --- | --- |
| **Purpose** | **Gene symbol** | **Forward primer** | **Reverse primer** |
| **RT-qPCR** | NTR1 | TTGCGCTTGATTGTTGATCTAG | CTCGAAACGAATAGCAAGTGAG |
|  | HSFA1B | TGCAAGAACTCGTCAGGTTAAGG | CTGGACTTTGAACAGCCTTTGC |
|  | HSFA1D | GGTAATGGTACGTCTACTACCG | CCGAAACATTTTCAGGAGTTGT |
|  | HSFA1E | TACTAAAGAGTATTGTCCGGCG | ACTTCCTCTTCGAGTCCAAATT |
|  | HSFA2 | TGAGAGGTTGAAGAGGGATCATGG | GCAAACTGCTGAACAAAGTTCGG |
|  | HSFA3 | GCTCTCTTTCTTGGCTAAGTTG | GTGGTGCTTGATGAACTTCTTT |
|  | HSFA4A | AGGGCTTGCTTTGAACCTATCG | AAGATTCTCCCAAATCGCTATCG |
|  | HSFA4C | ACGGAAAGAGTTTGAGCTGCAAG | TCGACCTGTTCTATGTGTGAACTGC |
|  | HSFA7A | TGAAGAACATCAAGAGGAGAAACCC | TGCATTGCTCTAGCCAGAAACG |
|  | HSFA7B | GGACCTTCTCAAAAGCATCAAG | CATCATTAGGACATGCCTCTCT |
|  | HSFB1 | GGATTTCGTAAAACTGTACCGG | ACCAGAATTAGACTCAGAAGGC |
|  | HSFB2A | TCTTCTCCGTGAGATCCAACGTC | ACCACCATTCCCAGTCGTCTTC |
|  | HSFB2B | TCCATCGAACTCTGGAGAAGAACAG | CTTTGTCATCTCTTTCCTCAATCGC |
|  | DREB2A | CATGTTTGATGTCGATGAGCTT | ATTCCGTAGTTGAGGCTTTGTA |
|  | HSP18.2 | GGTTGAGGTTGAAGACAAGAAC | TTCTCCGGTAACCTAAACCTTC |
|  | HSP17.4 | AGACACCTGAAGCGCATGTGTTC | TTCCTCCACCTTTGCATTCTCTG |
|  | HSP17.6II | TCCCCGAAGACCACAACAAC | TGCATATGCGTTAGGGTGCT |
|  | HSP22.0 | CTACTCCAGGCAGCTTGCTA | CTTGTGTCCCTCTCGAGTCC |
|  | HSP23.5 | CATCGGCTTTAGCTCTCAGG | GATGGTTCCTATCGACACCG |
|  | HSP60 | AAAAGATGGTGTTACTGTTGCC | CACCAGCAACATCATTAGTAGC |
|  | HSP70 | AGAGACGGGGTTTGATCCAG | TAGACAAGGGCTGCGTAAGT |
|  | HSP89.1 | GCGTATAGCTCCACTGCTTC | TCAAGTCCTTTCTCCAGCATCT |
|  | HSP90.7 | CGCTAGCGAAAACGTAGACG | GTGGGTTGATCTCGAGGACT |
|  | HSP101 | AGGTCATTCGTCGTGCTCAAGC | CTTCCCTTCTTTCCCACGAAGC |
|  | HSA32 | GCTGAACGGTGTTTAGAAGC | CAACCAACTTCGCATCAGATG |
|  | APX1 | GAGGTTTGACGCTGAGCAAG | TCAGCAAAAGAGATGGTAGGGA |
|  | ROF1 | GATGAAGAAAGGTGAAAACGCT | GTAGCATTCGCAGGAATAGTTG |
|  | BAG6 | CAATGTACTTTCAGATGCGGAG | TGTGCTCTTAACTGGCTCATTC |
|  | HOP3 | CCACCGCAATAACCCATTTCAC | CAACCGCTTCATCAAACTTGGAC |
|  | HTT1 | GTCAAATACCTCGCGTTCCC | TGGACTGAACAAATGCAGCC |
|  | HTT2 | AGAACATTGGGCACATGTGG | GGTCTGGACTGAACACATGG |
|  | HTT3 | CTATGGTAGCGGTGAATCTGAT | TTGTCTTCCTTCCCAGCATTAT |
|  | LUC | TGGAGAGCAACTGCATAAGG | GTTCACCTCGATATGTGCATCTGT |
|  | ILP1 | CCAAGAAGGCATTGAAAGACAA | GATAGCGAACTTTCAAGAGCTG |
|  | PRP43a | GGTGTTTCGAGAACTTTGTCAA | AAGCATGGCCTTTCTTATGTTG |
|  | PRP8 | GAAAGGTTTGCAGCTATACTCG | CCATTGATTGGCTTTGTCGTTA |
|  | TUB2 | GCCTTGTACGATATTTGCTTCAGGAC | CGGAGGTCAGAGTTGAGTTGAC |
| **AS RT-PCR** | HSFA2-AS | TGGGTTTCCTATGAGTCTTCAGC | ATCCCAGATCCTTGCTGATTCACA |
|  | HSFA4A-AS | AGCTTTATCCGCCAGCTTAACAC | CAACGGGTTTAACTGAGCTTGAAG |
|  | HSFA7A-AS | ACACTGACCACATCGTATCTTGGA | CGTCATGGCTTGGTGAAGATG |
|  | HSFA7B-AS | AAACCACATTGTGTCTTGGAACAG | TGAGACTGAGAGTAGTTAAGCGAAGG |
|  | HSFB1-AS | TTTAAGCAAAACGTATCAGCTAGTTG | CCGTCGTATGTCCGTCAACAG |
|  | HSFB2A-AS | TAGTTTCGTTCGTCAGCTCAACAC | CGAAGGAGCAACAACTGTTTGAT |
|  | HSFB2B-AS | AGCTTGTGGAAGATCCGGTTTAC | AAACTCCCAACGATCTGGAACTAC |
|  | HSP70-AS | TTCTCCAGAACCTGACGGAA | TGAGAAGCCTGGATCAAACCC |
|  | HSP89.1-AS | GTTAGTCGCCTCATGGACCT | AATTGTTGTCACCACCTGCAT |
|  | APX1-AS | TGAGGCCCACATGAAGCTTT | ATGTGTTCAAATCGCAACCC |
|  | HOP3-AS | CCTGAAGTTCAGAACATATTATCGGA | CCTGAACAATTCCGGCACTAAC |
|  | HTT2-AS | TCTCGTCTCTCTGTGTCTCCG | CTCTCCCTGCACATCTTACCTC |
|  | HTT3-AS | ATTTGCCTGTCAGATTCCTCG | CTCGGACTTATCCACTGCAAC |
|  | TUB2-AS | GCCTTGTACGATATTTGCTTCAGGAC | CGGAGGTCAGAGTTGAGTTGAC |
| **Semi-quantification** | NTR1-RT | TCTTCGCCATGGATGAGTATCAA | AACCGCCATCTTTCTGAGCC |
|  | STIPL2-RT | CAGACTAAGGAGGACGCTGT | GACCCGTTAGCAGGGAAATGT |
|  | ACTIN2-RT | AGATTCAGATGCCCAGAAGT | GCTCATACGGTCAGCGATA |
| **Genotyping** | Salk_073187 | CATAAGGTTGGCCAAGAGTCC | ACTGCCTTCCTCACCTTCTTC |
|  | Cs315805 | ACTTGTCATGCATTGCTTCC | ACCGTTGTGCATTCTATGAGG |
